# Unraveling the corrosion inhibition behavior of prinivil drug on mild steel in 1M HCl corrosive solution: insights from density functional theory, molecular dynamics, and experimental approaches

**DOI:** 10.3389/fchem.2024.1403118

**Published:** 2024-06-13

**Authors:** Abhinay Thakur, Ashish Kumar, Omar Dagdag, Hansang Kim, Avni Berisha, Deepak Sharma, Hari Om

**Affiliations:** ^1^ Division of Research and Development, Lovely Professional University, Phagwara, Punjab, India; ^2^ Nalanda College of Engineering, Department of Science, Technology and Technical Education, Government of Bihar, Bihar Engineering University, Nalanda, India; ^3^ Department of Mechanical Engineering, Gachon University, Seongnam, Republic of Korea; ^4^ Department of Chemistry, Faculty of Natural and Mathematics Science, University of Prishtina, Prishtina, Albania; ^5^ Deenbandhu Chhotu Ram University of Science and Technology, Murthal, Haryana, India

**Keywords:** corrosion inhibition, prinivil, mild steel, hcl, electrochemical analysis, mds

## Abstract

The deterioration of mild steel in an acidic environment poses a significant challenge in various industries. The emergence of effective corrosion inhibitors has drawn attention to studies aimed at reducing the harmful consequences of corrosion. In this study, the corrosion inhibition efficiency of Prinivil in a 1M HCl solution through various electrochemical and gravimetric techniques has been investigated for the first time. The results demonstrated that the inhibition efficiency of Prinivil expanded from 61.37% at 50 ppm to 97.35% at 500 ppm concentration at 298 K. With a regression coefficient (*R* ^2^) of 0.987, K_ads_ value of 0.935 and E_a_ value of 43.024 kJ/mol at 500 ppm concentration of inhibitor, a strong affinity of Prinivil for adsorption onto the metal surface has been significantly found. Scanning electron microscopy (SEM) and contact angle measurement analyses further support the inhibitory behavior of Prinivil, demonstrating the production of a defensive layer on the surface of mild steel. Additionally, molecular dynamics (MD) and Monte Carlo simulations were employed to investigate the stability and interactions between Prinivil and the metallic surface (Fe (1 1 0)) at the atomic level. The computed results reveal strong adsorption of Prinivil upon the steel surface, confirming its viability as a corrosion inhibitor.

## Graphical Abstract

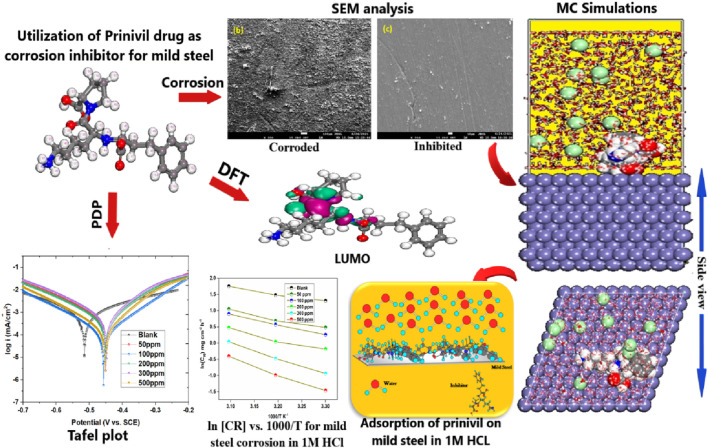

## 1 Introduction

Corrosion is a global problem that impacts numerous sectors globally, resulting in significant financial losses and environmental hazards (Lavanya and Machado, 2024; Rasheeda et al., 2024; Shoair et al., 2024). For example, in the oil and gas industry, the corrosion of pipelines and storage tanks may lead to oil spills, environmental contamination, and costly repairs. According to data from the U.S. Department of Transportation, corrosion-related incidents accounted for approximately 20% of all pipeline failures between 2002 and 2018, resulting in millions of gallons of oil being released into the environment (Al-gorair et al., 2024; Kaya et al., 2024). Additionally, corrosion-related maintenance and repair costs in the oil and gas sectors are estimated to exceed billions of dollars annually. Similarly, in the marine industry, the corrosion of ship hulls and offshore structures not only compromises their structural integrity but also increases maintenance and operational expenses (Althobaiti et al., 2023; Betti et al., 2023; Ozoemena et al., 2023). In infrastructure and construction projects, the deterioration of steel reinforcement in concrete frameworks can lead to structural failures, requiring extensive repairs and replacements (Al-gorair et al., 2024; Li et al., 2024). Mild steel, with its widespread use in several applications including construction, automotive, infrastructure, transportation, etc., is particularly susceptible to corrosion in acidic environments (Cui et al., 2024; Dahmani et al., 2024). This vulnerability is a global concern, affecting industries in diverse geographical regions. For instance, in coastal areas with high levels of airborne salts, such as coastal cities or offshore installations, mild steel corrosion could be fastened by the inclusion of chloride ions (Martinović et al., 2023; Shoair et al., 2024). In industrial environments, such as chemical plants and manufacturing facilities, exposure to acidic solutions or fumes can lead to severe corrosion of mild steel equipment and structures. These examples highlight the global nature of the corrosion problem and the need for effective corrosion mitigation strategies.

To combat corrosion, the development of corrosion inhibitors has gained significant attention globally. Organic compounds, including drug molecules, have evolved as viable corrosion inhibitors due to their varied chemical structures and potential for adsorption onto metal surfaces (Andrés Coy-Barrera et al., 2023; Njoku et al., 2023a; Nie et al., 2023a). This approach has been explored in various regions. For instance, European researchers have investigated the corrosion inhibition potential of organic compounds, including drug molecules, for applications such as the protection of cultural heritage structures or the preservation of historical artifacts. In North America, studies have focused on the usage of organic corrosion inhibitors for oil and gas pipelines and infrastructure projects. In Asia, particularly in countries with high industrial activity, investigations have been carried out on the corrosion inhibition potency of organic compounds for applications in chemical plants and marine environments (Cui G. et al., 2023; Uzah et al., 2023a).

Drug molecules often possess functional moieties including amine, carboxyl or hydroxyl moieties, which can readily adsorb upon metallic surfaces (Njoku et al., 2023b; Hadisaputra et al., 2023; Verma et al., 2023). The emergence of a barrier-like coating of protection is made possible by this adsorption procedure. Which inhibits corrosive chemicals from accessing the metallic surface and slows down the rate of corrosion (Hameed et al., 2021). Researchers in different parts of the world have explored the corrosion inhibition characteristics of drug molecules including metformin (Onyeachu et al., 2021), ibuprofen (Tasić et al., 2019), and indomethacin (Golgovici et al., 2020) on various metals, including mild steel. For instance, Onyeachu et al. (Onyeachu et al., 2021) have demonstrated the corrosion inhibition potential of antidiabetic drugs, such as metformin, on mild steel in an HCl medium. Several empirical investigations demonstrated that metformin demonstrated a significant corrosion inhibition efficiency of 90% at 200 ppm, exhibiting a noticeable elevation in the inhibition efficacy (I.E., %) and reduction in the corrosion rate (C_R_), as the concentration of the inhibitor increased. The sorption of metformin onto the mild steel surface was attributed to the formation of a protective film, which effectively prevented corrosive ions from attacking the substrate. Similarly, investigations into the corrosion inhibition characteristics of nonsteroidal anti-inflammatory drugs (NSAIDs), like ibuprofen (Tasić et al., 2019) and indomethacin (Golgovici et al., 2020), have also demonstrated their effectiveness in mitigating mild steel corrosion in corrosive environments. These drugs exhibited strong inhibition performance, leading to a considerable reduction in the C_R_ and an enhancement in the, I.E., % with rising inhibitor concentration. The main mechanism accountable for the corrosion prevention characteristics of ibuprofen and indomethacin was found to be the production of a defensive coating via their adsorption on the metallic surface.

**FIGURE 1:**
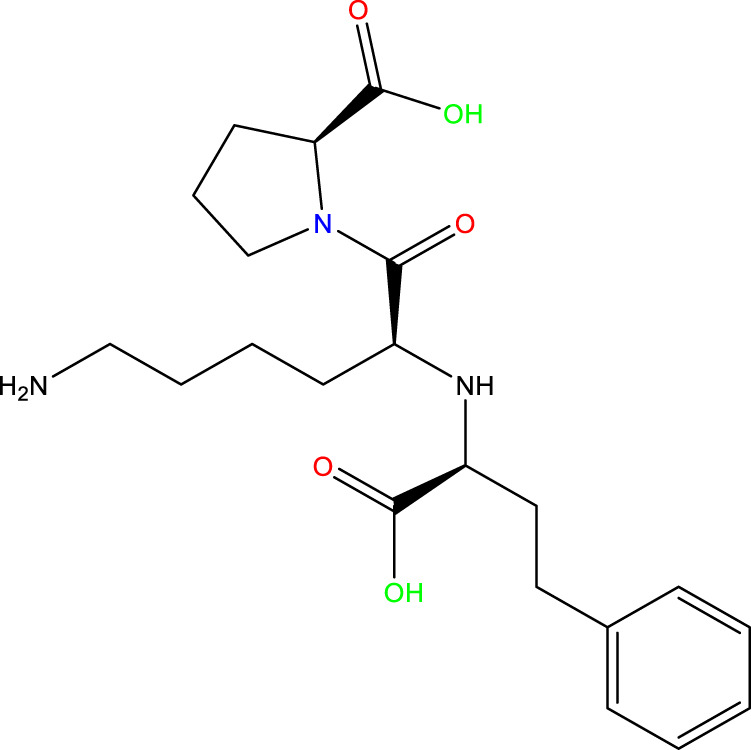
Chemical configuration of the Prinivil molecule (Molecular weight = 405.5 g/mol).

Given the success of drug molecules as corrosion inhibitors on several metals, such as mild steel, there is a need to explore the potential of other drug molecules in corrosion prevention. Prinivil, a well-known drug belonging to the class of angiotensin-converting enzyme (ACE) inhibitors, exhibits promising potency as a corrosion inhibitor owing to its chemical configuration and functional groups (Dong et al., 2023a; Rinky et al., 2023). Specifically, Prinivil contains carboxylic acid and amine groups (as illustrated in Figure 1), which are known to facilitate linkages with metallic surfaces and contribute to the development of a defensive layer. In acidic environments, such as a 1 M HCl solution, mild steel is susceptible to corrosion. The corrosive attack occurs through the dissolution of iron ions from the metallic surface, culminating in the degradation of the steel (Chukwunyere and Egbosiuba, 2023). However, the existence of an effective corrosion inhibitor like Prinivil can mitigate this process by forming a defensive layer upon the substrate, thus preventing corrosive entities from accessing the metallic surface. Prinivil molecules adsorb upon the metallic surface through interactions between their functional groups (carboxylic acid and amine) and the metal atoms (Swathi et al., 2023a; Ikeuba et al., 2023). This adsorption process is crucial for establishing a defensive layer against corrosive entities. Upon adsorption, Prinivil molecules may undergo chemical reactions or rearrangements to form a stable and compact film upon the metallic surface. This layer behaves as a physical obstacle, reducing the diffusion of corrosive species including HCl molecules or chloride ions to the underlying metal. Moreover, the protective film formed by Prinivil inhibits both cathodic and anodic reactions involved in the corrosion phenomenon (Fawzy et al., 2023a). It may suppress the reduction of protons (H⁺ ions) at cathodic sites and/or the oxidation of iron ions (Fe^2^⁺) at anodic sites, thereby retarding the overall C_R_. Thus, the corrosion inhibition process of Prinivil upon mild steel involves the adsorption of its functional groups upon the surface, the emergence of a defensive layer, and the inhibition of corrosive reactions.

The objective of this research is to unveil the corrosion inhibition potential of Prinivil drug molecule on mild steel in a 1 M HCl corrosive media. By employing a blend of empirical and computational methods, this research intends to comprehensively investigate the inhibitory phenomenon of Prinivil and evaluate its potential as a corrosion inhibitor (Deyab et al., 2023; Sayed et al., 2023; Yeganeh et al., 2023). Experimental analysis will involve electrochemical measurements, including electrochemical impedance spectroscopy (EIS) and potentiodynamic polarization (PDP), to assess the corrosion inhibition efficacy of Prinivil on mild steel. Computational techniques, including Monte Carlo (MC), Molecular dynamics (MD) simulations and density functional theory (DFT) calculations, will offer insights into the adsorption characteristics, stability, and interactions between Prinivil and the surface of the mild steel (Ozoemena et al., 2023). Ultimately, the findings from this study will contribute to the existing body of literature on drug molecules as potent corrosion inhibitors for several metals including mild steel and may contribute to the emergence of novel corrosion inhibitors, improving the durability and safety of mild steel structures in aggressive acidic environments.

## 2 Experimental methods

### 2.1 Materials

The mild steel sample utilized for corrosion studies was characterized by a composition with a purity percentage of Mn-1.27, C-0.08, Si-0.01, P-0.02, and Fe-98.62%. The dimensions of the mild steel specimen employed for weight loss corrosion measurements were specified as 1 cm^2^, providing a standardized size for testing purposes (Chukwunyere and Egbosiuba, 2023; Mubarak et al., 2023). Before conducting corrosion tests, the sample underwent thorough preparation to ensure consistency and reliability of results. This preparation involved polishing the surfaces using emery papers of varying grit sizes ranging from 400 to 2000. The use of different grit sizes allowed for the gradual refinement of the surface, ultimately achieving a mirror-like finish. Furthermore, the cleaning procedure employed for the mild steel specimens adhered to the guidelines outlined in ASTM G-31 practice (Abdel-Hameed et al., 2023; Zobeidi et al., 2023).

### 2.2 Test solutions

In this experimental setup, electrolytes were prepared both with and without the inclusion of several concentrations of inhibitor in 1 M HCl media. Specifically, concentrations of 50, 100, 200, 300, and 500 parts per million (ppm) of inhibitor were employed. By introducing the inhibitor into this solution, researchers aimed to assess its effectiveness in mitigating or inhibiting the deleterious outcomes of the acidic environment on the metallic substrate (Alao et al., 2023; Betti et al., 2023). By varying the concentration of inhibitor in 1 M HCl solution, researchers could explore how different levels of acidity and inhibitor influence the corrosion inhibition characteristics of the inhibitor. This experimental design allows for a comprehensive evaluation of the inhibitor’s performance across a range of corrosive conditions, providing valuable insights into its efficacy under various scenarios.

### 2.3 Preparation of the electrode surface

The surface preparation of the electrodes involved the fabrication of working electrodes utilizing the same metal composition as described previously in Section 2.1. These electrodes were intended for various electrochemical analysis, including EIS and PDP. In order to accomplish this, metallic rods were cut (5 cm in length), and one end was adhered to copper (Cu) wire to ensure electrical conductivity (Afshari et al., 2023; Ojo et al., 2023). The exposed portion of the electrode was then coated with epoxy resin, leaving the lower section uncovered, creating a 1 cm^2^ accessible interfacial region. This layout permitted researchers to partially expose the metallic sample to the acidic media, facilitating further analysis and experimentation.

### 2.4 Weight loss (WL) measurement

The weight loss experiment involved immersing a mild steel sample with a defined surface region of 1 cm^2^ into a 1 M HCl media, both with and without varying concentrations of inhibitor (50, 100, 200, 300, and 500 ppm). Each specimen was initially weighed utilizing an analytical balance having a sensitivity of 0.0001 g before submersion in the corrosive medium (100 mL of 1 M HCl), along with the prinivil solution necessary for the experiment (Alimohammadi et al., 2023; Yousif et al., 2023). The dipping period ranged from 15 to 60 min at temperatures ranging from 303 K to 323 K, controlled using a thermostat to ensure stability. Upon removal from the solution, the specimens underwent thorough cleaning with acetone and water, followed by air-drying and a second weighing to determine inhibition efficiency (IE_wl_), surface coverage (ϴ), and C_R_. Fresh solutions were prepared for each trial, and the experiment was repeated thrice to ensure the accuracy and reliability of the outcomes. Eqs 1, 2 were employed to calculate IE_wl_ and ϴ, respectively:

$${IE}_{wl}=\frac{W_{0}-W_{i}}{W_{0}}\times 100$$ (1)

$$\theta =\frac{{\;IE}_{wl}}{100}$$ (2)

W_0_ and W_i_ represent the weight loss measurements before and after the inclusion of the inhibitor, respectively. Additionally, the C_R_ of the mild steel was determined using Eq. 3:

$$C_{R}\left(mm/y\right)=\frac{87.6\times w}{at\;D}$$ (3)

w denotes the weight loss experienced by the mild steel due to corrosion (in mg), t denotes the duration of exposure (in hours), a denotes the surface region of the sample (in cm^2^), and D represents the density of the metallic sample.

### 2.5 Electrochemical measurements

To assess the corrosion inhibition efficiency of Prinivil, electrochemical analysis including EIS and PDP were conducted utilizing the Metrohm Autolab NOVA 2.1.7 electrochemical apparatus. The experiments were performed in a single-chamber electrochemical cell designed for evaluating various flat samples (Golshani et al., 2023; Simović et al., 2023). This cell incorporated a three-electrode system: mild steel as a working electrode having a 1 cm^2^ accessible area, a Pt rod (99.9% purity) serving as the counter electrode and a reference electrode (saturated calomel electrode (SCE)). To assure a stable condition prior to commencing the evaluation, the working electrode was submerged in 1 M HCl solution both in the exclusion and inclusion of Prinivil for around an hour at the Open Circuit Potential (OCP). Subsequently, polarization curves were scanned at a rate of 1 mV/s by varying potentials around the recorded OCP after a 1-h immersion period (Akkoç et al., 2023; Shi et al., 2023).

For EIS analysis, a sinusoidal voltage with an amplitude of 0.005 V was applied, covering a frequency range from 100 kHz to 10 mHz. By extending the linear Tafel portions of the cathodic and anodic curves to the corrosion potential (E_corr_), corrosion current densities (i_corr_) were computed. To ensure reliability, all electrochemical experiments were carried out at room temperature without disturbance. The polarization variables were attained employing the following equation:

$${IE}_{pdp}=\frac{i_{corr-\;i_{\prime corr}}}{i_{corr}}\times 100$$ (4)

In this context, i_corr_ and i_’corr_ resemble the corrosion current density in the exclusion and inclusion of the inhibitor. The charge transmission values were employed to compute the, I.E.,% using the eqn. provided below:

$${IE}_{eis}=\left[R{ct}^{i}-\frac{R{ct}^{0}}{R{ct}^{i}}\right]\times 100$$ (5)

Herein, Rct^0^ and Rct^i^ represent the charge transfer resistance in the exclusion and inclusion of the inhibitor, correspondingly.

### 2.6 Surface morphology analysis

#### 2.6.1 Scanning electron microscopy (SEM)

SEM is a potent imaging method utilized to evaluate, at extreme magnification, the surface shape and characteristics of materials. SEM was used in this work to evaluate the mild steel specimens’ surface features and morphological alterations (Swathi et al., 2023a; Chukwunyere and Egbosiuba, 2023). Once the samples were exposed to the corrosive atmosphere for 6 hours, both with and without Prinivil, they were meticulously prepped and positioned on SEM stubs. The surface topography was acquired by the SEM visuals, which made it possible to assess surface pitting, corrosion byproducts, and the expansion of a defensive layer upon the metallic surface. The effects of Prinivil as a potent corrosion inhibitor were demonstrated visually by the high-resolution SEM visuals.

#### 2.6.2 Energy-dispersive X-ray (EDX) analysis

The samples were prepared by immersing mild steel specimens in 1 M HCl solution with and without 500 ppm of Prinivil for 6 h (Sharma et al., 2022). EDX analysis was conducted using an SEM equipped with an EDX detector. The EDX spectra were collected from representative areas of the sample surfaces to identify the elemental composition of the corrosion products (Kaur et al., 2022; 2023; Nidhi and Kaur, 2022). The obtained EDX spectra were analyzed to determine the presence and relative abundance of elements such as Fe, N, C, O, and any other relevant elements.

#### 2.6.3 Contact angle (CA) measurement

CA measurement is a procedure utilized to evaluate the wettability and surface energy of materials. In the context of corrosion mitigation, CA measurements offer insights into the emergence of a hydrophobic defensive layer upon the surface of mild steel. A contact angle goniometer was utilized in this research to measure the contact angle formed between the corrosive solution and the metallic surface (Andrés Coy-Barrera et al., 2023; Yadav et al., 2023a; Martinović et al., 2023). The contact angle is impacted by the interfacial forces between the liquid, solid, and gas phases, and a higher contact angle indicates a less wettable surface. It suggests the development of a hydrophobic layer that can act as an obstacle against corrosive species. The CA measurements aid in the understanding of the potential formation of a protective layer facilitated by Prinivil.

### 2.7 Computational analysis

Computational analysis, including DFT, MC and MD simulations, played a pivotal role in providing detailed perspectives into the corrosion inhibition potential of Prinivil on mild steel at the molecular and atomic levels (Abeng et al., 2023). These computational approaches allowed for a comprehensive understanding of the interactions between Prinivil molecules and the surface of mild steel, shedding light on the underlying mechanisms of corrosion inhibition. Furthermore, the reactivity of organic compounds and their ability to prevent the corrosion of metals have long been studied using quantum chemical techniques (Nie et al., 2023b; Uzah et al., 2023b; Qureshi et al., 2023). We performed DFT computations for this objective using the Dmol3 module that has been embedded in the Biovia Materials Studio software. Geometry refinements have been performed using the Generalized Gradient Estimation using the Double Numerical plus d-functions (DNP) and M06l. Utilizing a 13.214 Fe thickness (c-axis), the dynamics between the metallic surface and the prinivil (low pH) and prinivil molecules can be analyzed in a hypothetical corrosive condition. There were three dimensions to the plate layout: 24.823752 × 24.823752×53.241658 + 40 Å vacuum coating at the C axis which includes: 800 H_2_O molecules, 1 inhibitor molecule, 10 hydronium ions and 10 chloride ions (Cui G. D. et al., 2023; Hou et al., 2023). The geometry of the simulated chambers was adjusted before the MD phase utilizing the Forcite component that is integrated within the Biovia package (Gnedenkov et al., 2023; Kumar et al., 2023). The standard NVT assembly was used for MD, which was run at 298 K and 1,000 ps (1 fs duration phase) of simulation time. The condensed phase systems MC and MD were derived using the COMPASS force field (version 1.0).

Various quantum chemical variables such as global hardness (*η*), electronegativity (*χ*), electronic affinity (*A*), ionization potential (*I*) and softness (σ), E_LUMO_, E_HOMO_ were calculated by employing Eqs 6–12 to attain the reactivity of the corrosion inhibitor.

$$△E=E_{HOMO}-\;E_{LUMO}$$ (6)

$$I=-E_{HOMO}$$ (7)

$$A=-E_{LUMO}$$ (8)

$$\eta =\left(\frac{I-A}{2}\right)$$ (9)

$$\chi =\left(\frac{I+A}{2}\right)$$ (10)

$$\sigma =\frac{1}{\eta }$$ (11)

$$\Delta N=\frac{\chi_{Fe}-\chi_{Inh}}{2\left(\eta_{Fe}-\eta_{inh}\right)}$$ (12)

Herein, η_inh_ and χ_inh_ represent the hardness and electronegativity of the inhibitor. Values of ΦFe (110) = 4.82 eV and ηFe (110) = 0eV were utilized to calculate the ΔN.

Moreover, Fukui indices were employed to ascertain the sites within the molecules where electron donation and acceptance occur. Furthermore, Fukui functions coupled with Mulliken population analysis (NPA) were utilized to identify the specific regions of local reactivity within the investigating inhibitor.

$$\text{Nucleophilic attacks}\to \;{ƒ}_{k}^{+}=P_{k}\left(N+1\right)\;–\;P_{k}\left(N\right)$$ (13)

$$\text{Electrophilic attacks}\to \;{ƒ}_{k}^{-}=P_{k}\left(N\right)\;–\;P_{k}\left(N-1\right)$$ (14)

Here, *P*_*k*_(*N*), *P*_*k*_(*N+1*)and *P*_*k*_(*N-1*) represent the neutral, anionic and cationic Mulliken populations of the inhibitor molecules.

## 3 Result and discussion

### 3.1 Weight loss measurement

The weight loss assessment method is frequently employed to analyze the corrosion inhibition efficacy of inhibitors in various metals and alloys (Thakur et al., 2019; 2020; Thakur et al., 2023 R. C.; Nidhi et al., 2023; Pathania et al., 2023; Rana et al., 2023). By measuring the weight loss of a specimen as a consequence of the corrosion phenomena, this method offers important information about the C_R_ and the efficacy of corrosion inhibitors (Dong et al., 2023b; Bijapur et al., 2023; Chowdhury et al., 2023). In our investigation, we conducted a weight loss evaluation following the immersion of samples in a 1M HCl media for 24 h as depicted in Supplementary Figure S1. The impact of temperature on the C_R_ and, I.E., % can be observed by analyzing the data in Table 1. With the rising temperature from 303 K to 323 K, the corrosion rate generally tends to increase for both the blank solution and solutions with inhibitor. For example, at 303K, the corrosion rate for the blank solution ranges from 0.23 mm/y (500 ppm) to 3.68 mm/y (blank), while at 323 K, it ranges from 0.67 mm/y (500 ppm) to 5.76 mg/cm^2^h (blank). This increase in corrosion rate with temperature indicates that higher temperatures accelerate the corrosion process, making the environment more aggressive.

**TABLE 1:** Gravimetric outcomes such as *C*_*R*_, *IE* %, and *ϴ* at 303 K, 313 K, and 323 K for mild steel in 1 M HCl in the exclusion and inclusion of the prinivil.

Temperature (K)	Concentration (ppm)	*C*_*R*_ (mm/y)	IE %	*ϴ*
Blank	0	3.68	-	-
303	50	1.62	55.98	0.560
	100	1.29	64.95	0.650
	200	0.83	77.45	0.775
	300	0.39	89.40	0.894
	500	0.23	93.75	0.938
Blank	0	4.38	-	-
313	50	1.98	54.80	0.548
	100	1.76	59.82	0.598
	200	1.04	76.26	0.763
	300	0.62	85.85	0.859
	500	0.37	91.53	0.915
Blank	0	5.76	-	-
323	50	2.85	50.52	0.505
	100	2.45	57.47	0.575
	200	1.61	72.05	0.721
	300	1.04	81.94	0.819
	500	0.67	88.37	0.884

**FIGURE 2:**
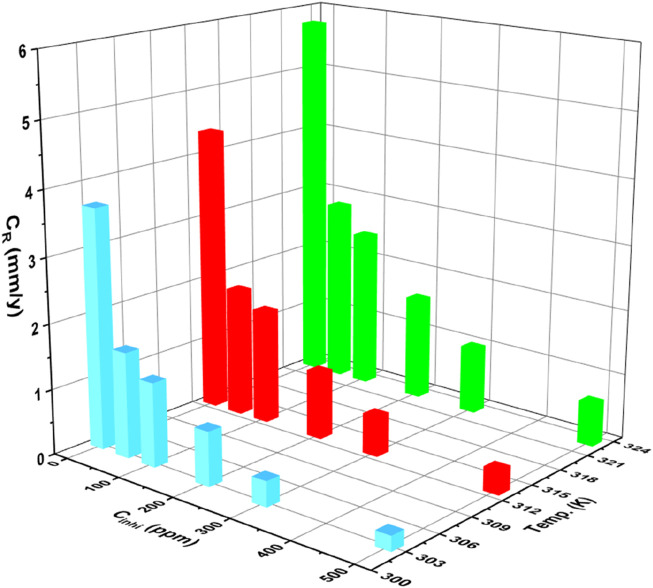
Bar chart illustrating the variation of corrosion rate with the inhibitor concentration at different temperatures.

Despite the elevation in C_R_ with temperature, the inhibitor demonstrates a consistent trend of reducing the corrosion rate across different temperatures. At all temperatures (303, 313, and 323 K), the inclusion of the inhibitor leads to lower C_R_ compared to the blank solution. For instance, at 303 K, the inhibition efficiency ranges from 55.98% (50 ppm) to 93.75% (500 ppm), indicating effective corrosion inhibition even at higher temperatures (Figure 2). This suggests that the inhibitor maintains its effectiveness in reducing corrosion rates, even in more aggressive environments at higher temperatures (Swathi et al., 2023b; Marashi-Najafi et al., 2023). Despite the challenges posed by elevated temperatures, the inhibitor demonstrates its effectiveness in mitigating corrosion across different temperature ranges. This underscores the potential of the inhibitor for practical applications where temperature variations are encountered, highlighting its role in providing corrosion protection in challenging environments.

### 3.2 Electrochemical analysis

#### 3.2.1 Potentiodynamic polarization (PDP)

**FIGURE 3:**
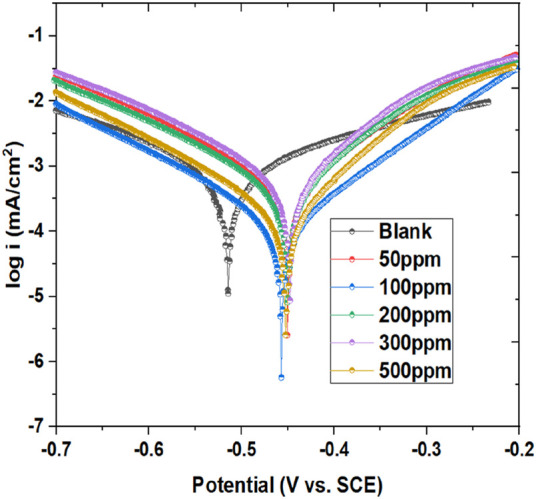
Tafel plot showing mild steel’s corrosion inhibition in a blank medium exposed to 50–500 ppm of inhibitor at 298 K.

PDP is a key electrochemical methodology utilized to analyze corrosion behavior and, I.E., % in metals exposed to corrosive environments. By measuring current density as the metal is subjected to varying potentials, PDP generates polarization curves. These curves help determine E_corr_ and i_corr_, crucial parameters for understanding corrosion rates. PDP also assesses the potential of corrosion inhibitors by comparing curves with and without inhibitors (Merimi et al., 2023a; Dong et al., 2023b). In current PDP measurements, the metal specimen was exposed to a range of elevated concentrations of inhibitor in a corrosive solution. The E_corr_, i_corr_, and Tafel slopes for both anodic (β_a_) and cathodic (β_c_) reactions were evaluated from the linked polarization variables, as detailed in Table 2. The polarization plots have been illustrated in Figure 3. As the prinivil dosage elevated from 50 ppm to 500 ppm, the corrosion inhibition mechanism underwent a transition, primarily owing to the enhanced adsorption of inhibitor molecules on the metal surface (Hadisaputra et al., 2023; Motawea and Melhi, 2023). At lower concentrations, the inhibitor molecules predominantly adsorb on the anodic sites of the metallic surface, where they hinder the oxidation reactions responsible for corrosion. However, as the inhibitor dosage rises, more inhibitor molecules adsorbed not only on the anodic sites but also on the cathodic sites of the metallic surface. This increased adsorption on both anodic and cathodic sites leads to a shift in the corrosion inhibition mechanism towards the cathodic side. The inhibitor molecules form a protective layer over the metal surface, inhibiting both anodic and cathodic corrosion processes effectively. This shift in the mechanism highlights the importance of inhibitor concentration in modulating the corrosion inhibition efficiency and the overall protection of the metallic surface against corrosion. The, I.E., % of the inhibitor was calculated using Equation 4.

**TABLE 2:** Tafel parameters for the mild steel’s corrosion inhibition in corrosive media exposed to 50–500 ppm of inhibitor at 298 K.

Conc. (ppm)	-E_corr_ (mV vs SCE)	i_corr_ (µA)	βa (mV/dec)	-βc (mV/dec)	C_R_ (mm/yr)	IE%	χ^2^
Blank	513.81	718.41	189.46	145.56	17.185	-	2.175E-07
50	440.85	362.06	174.28	109.23	8.0451	49.60	5.7845E-08
100	446.26	266.08	163.226	124.9	6.8448	62.96	2.4757E-07
200	446.37	218.88	145.693	128.3	4.0307	69.53	5.2485E-07
300	447.98	151.52	127.373	118.16	1.9607	78.90	1.1475E-06
500	451.49	92.77	109.26	115.4	1.3685	87.08	1.0145E-06

Figure 3 and Table 2 demonstrate the potency of prinivil in mitigating corrosion of mild steel, with higher concentrations culminating in greater, I.E., % and surface coverage. As observed, the concentration of prinivil, ranging from 0 ppm (blank) to 500 ppm, indicates the varying dosages of the inhibitor tested in the experiment. As the concentration increases, there is a noticeable shift in the -E_corr_ towards less negative potentials, decreasing from 513.81 mV (blank) to 451.49 mV (500 ppm). This shift suggests that higher concentrations of prinivil effectively suppress corrosion initiation by developing a defensive barrier on the metallic surface which means that the inhibitor has more effect on the anode process than the cathode. It could be stated that the studied inhibitor is a mixed inhibitor (because the values of E_corr_ displacement for all concentrations do not exceed ±85 mV) with more effect on the anodic process. Concurrently, the i_corr_ decreases from 718.41 µA (blank) to 92.77 µA (500 ppm), indicating a drop in the rate of corrosion with increasing inhibitor dosage. The drop in i_corr_ signifies the effectiveness of prinivil in slowing down the corrosion process on the mild steel surface. The β_a_ and β_c_ values also exhibit a decreasing trend with increasing prinivil concentration (Abeng et al., 2023). The decrease in β_a_ (from 189.46 mV/dec to 109.26 mV/dec) and β_c_ (from 145.56 mV/dec to 115.4 mV/dec) suggests a reduction in the rates of both cathodic and anodic corrosion phenomenon. This reduction in corrosion rates is further supported by the decrease in C_R_ values, which decrease from 17.185 mm/yr (blank) to 1.3685 mm/yr (500 ppm). Furthermore, the, I.E.,% increases from not applicable (blank) to 87.08% (500 ppm), indicating a higher degree of corrosion inhibition achieved with higher concentrations of prinivil. This increase in inhibition efficiency is accompanied by a corresponding increase in surface coverage (χ^2^) values, which increase from 2.175E-07 (blank) to 1.0145E-06 (500 ppm). The higher surface coverage demonstrates that more inhibitor molecules adsorb onto the metallic surface, leading to enhanced corrosion protection. The observed trends in E_corr_, i_corr_, Tafel slopes, C_R_, I.E., %, and surface coverage collectively indicate the corrosion inhibition potential of prinivil and its ability to protect mild steel surfaces from corrosive environments.

#### 3.2.2 Electrochemical impedance spectroscopy (EIS)

**FIGURE 4:**
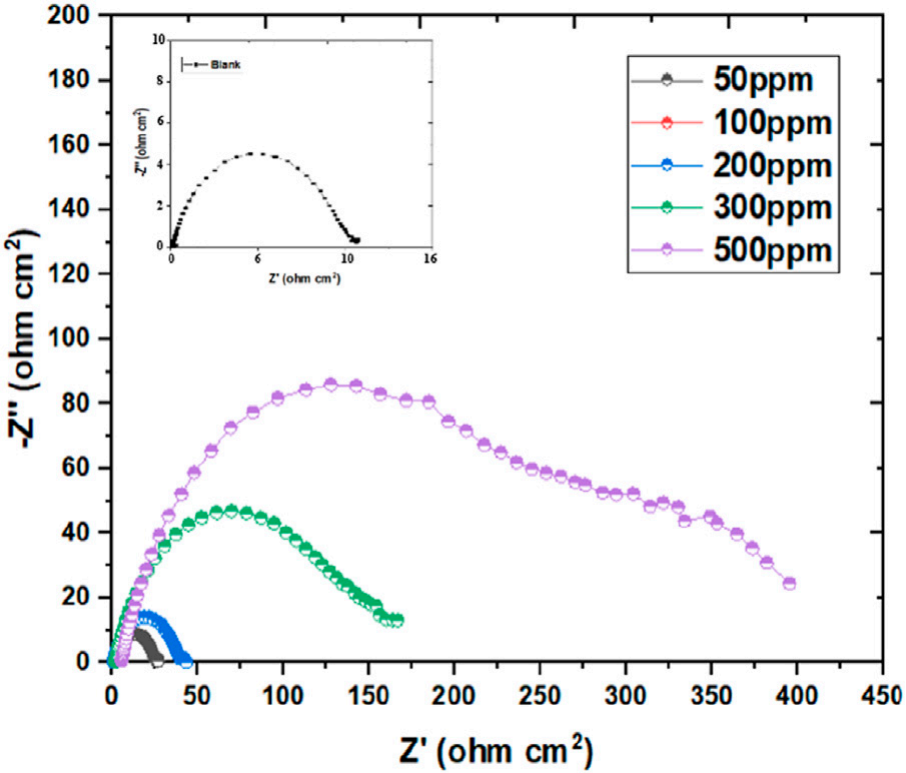
Nyquist graph for mild steel’s corrosion inhibition in blank media exposed to 50–500 ppm of inhibitor at 298 K.

The investigation of Nyquist plots offers valuable information about the impedance behavior of corrosion inhibitors in acidic environments. In this study, Nyquist plots were obtained for inhibitor dosage ranging from 50 ppm to 500 ppm, alongside corresponding blank samples, providing critical information on the inhibition efficiency and impedance characteristics. The Nyquist plots revealed distinct differences between the inhibitor-containing samples and the blank samples. As depicted in Figure 4, at lower inhibitor concentrations (50 ppm and 100 ppm), the plots exhibited intermediate impedance values, indicating a moderate inhibition effect on the corrosion process (Dong et al., 2023b). The presence of inhibitors led to an elevation in the R_CT_ compared to the blank samples, suggesting a reduction in the C_R_. As the inhibitor concentration increased (200 ppm, 300 ppm, and 500 ppm), further enhancements in impedance behavior were observed. The Nyquist plots showed higher R_CT_ values for the inhibitor-containing samples, signifying a more pronounced inhibition effect. This increase in impedance suggested a greater hindrance to the corrosion process, resulting in improved corrosion protection. Moreover, the comparison between the R_CT_ values of inhibitor-containing samples and blank samples highlighted the effectiveness of the inhibitors in mitigating corrosion (Swathi et al., 2023a). The inhibitor-containing samples consistently exhibited higher R_CT_ values, indicating superior corrosion inhibition performance compared to the blank samples. Additionally, as inhibitor concentration increases, the capacitive circle in Nyquist plots continues to expand, which indicates improved corrosion, I.E., % and related alterations to the electrochemical dynamics of the system (Pour-Ali et al., 2023a). This phenomenon may be explained by the inhibitor molecules adhering to the metallic surface and creating a barrier that prevents corrosion. At lower inhibitor concentrations, the capacitive loop exhibited moderate enhancement, indicating a partial inhibition effect. As the inhibitor concentration increased, the capacitive loop became more pronounced, signifying a stronger inhibition effect and improved corrosion protection. This behavior is indicative of the inhibitor’s ability to hinder the electrochemical reactions responsible for corrosion, such as metal dissolution and oxygen reduction.

The mechanism underlying the increasing capacitive loop involves the emergence of an adsorbed inhibitor coating on the metallic substrate (Huang et al., 2023). This layer behaves as a physical obstacle, preventing direct contact between the corrosive environment and the metallic substrate. Additionally, the inhibitor molecules might offer chemical reactions with the metallic substrate, developing a defensive layer that further impedes corrosion processes. Furthermore, the elevation in the capacitive loop corresponds to an elevation in the R_CT_ observed in Nyquist plots. This elevation in R_CT_ suggests a lessening in the rate of electron transfer reactions at the metal-electrolyte interface, culminating in decreased corrosion rates. Additionally, the appearance of a diffusion tail at high inhibitor concentrations (300 and 500 ppm) in Figure 4 indicates a notable alteration in the transport dynamics of ions or molecules within the electrolyte solution. This phenomenon typically arises due to the presence of the inhibitor, which impedes the free movement of species through the solution. The inhibitor formed a protective layer on the electrode surface or modified the transport properties of the electrolyte solution. The formation of a protective layer, often initiated by the adsorption of inhibitor molecules onto the electrode surface, creates a barrier that restricts the diffusion of ions or molecules toward the electrode interface. This protective layer serves as a shield against corrosive species, thereby reducing the rate of corrosion. Additionally, the inhibitor might induce changes in the transport properties of the electrolyte solution itself. For instance, the presence of inhibitor molecules can alter the viscosity or conductivity of the solution, affecting the mobility of ions and molecules within the electrolyte. As a result, the diffusion of species towards the electrode surface becomes hindered, leading to the observed diffusion tail in the EIS data.

**FIGURE 5:**
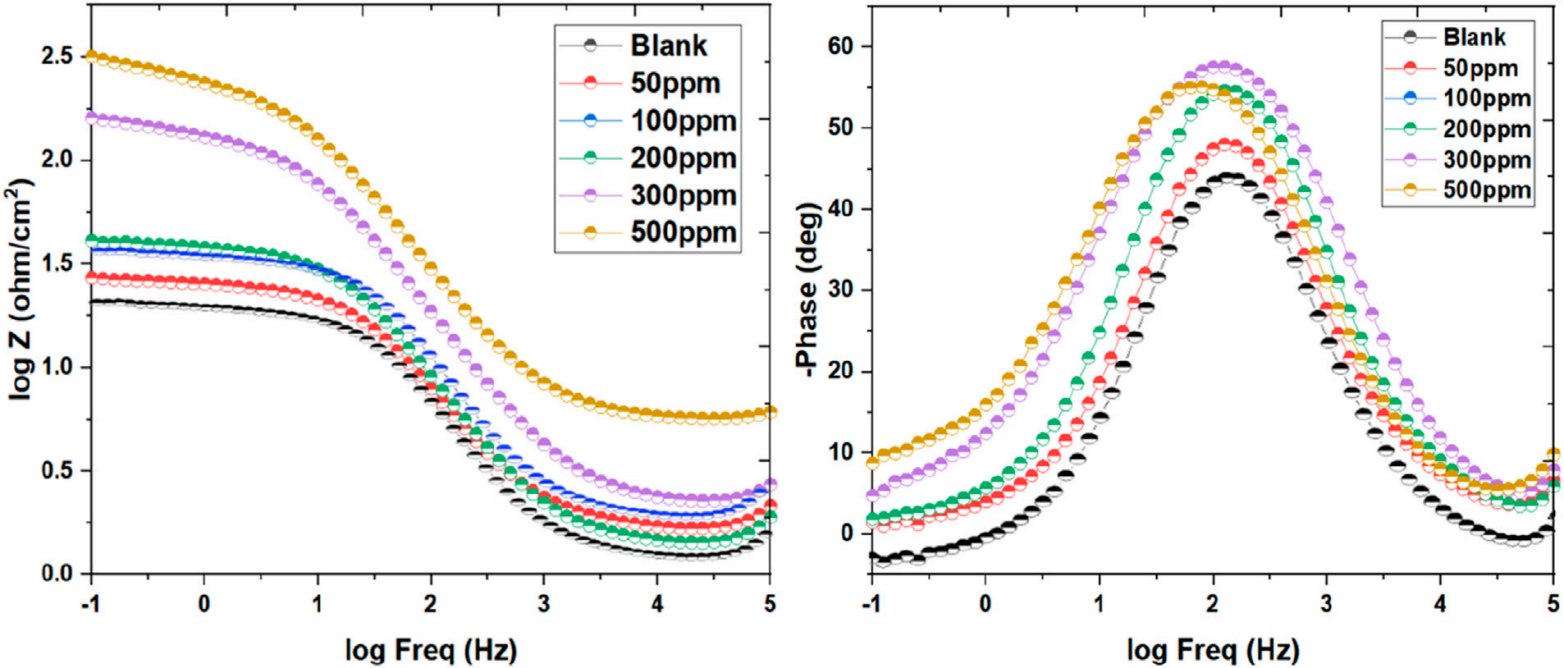
Bodes plot for the mild steel’s corrosion inhibition in blank media exposed to 50–500 ppm of inhibitor at 298 K.

The examination of capacitive behavior through Bode plots offers valuable insights into the efficacy of corrosion inhibitors across different concentrations. In this study, Bode plots (Figure 5) were generated for inhibitor dosage varying from 50 ppm to 500 ppm in an acidic environment of 1 M HCl. At lower inhibitor concentrations (50 ppm and 100 ppm), the Bode plots exhibited deviations from ideal behavior, with phase angles diverging notably from the desired 90°. This divergence suggested a less favorable capacitive response and hinted at potential limitations in corrosion inhibition effectiveness at these concentrations (Tolulope Loto, 2023; Wang et al., 2023). However, as the inhibitor concentration increased (200, 300, and 500 ppm), notable improvements in capacitive behavior were observed. The curves began to approach the desired characteristics, with phase angles aligning more closely with the ideal 90°. This trend indicated a more favorable capacitive response and suggested enhanced corrosion, I.E.,% at higher inhibitor dosage. The optimization of capacitance at intermediary frequencies holds significant implications for corrosion prevention strategies. By achieving a more optimal capacitive behavior, inhibitors can better protect metallic surfaces from corrosive attacks in acidic environments. For the fitting of the acquired data an equivalent circuit used was used (Supplementary Figure S2). These findings underscore the importance of concentration-dependent effects on corrosion inhibition efficacy and provide valuable guidance for the emergence of more potent corrosion control measures.

Table 3 deliberates an overview of the corrosion mitigation effectiveness of mild steel in a highly corrosive 1 M HCl solution when subjected to varying concentrations of inhibitor (50–500 ppm) at 298 K. The inclusion of electrochemical parameters such as the constant phase element (CPE.Yo), exponent (n), solution resistance (R_s_), polarization resistance (R_p_), and charge transfer resistance (R_ct_) provide insightful information about the inhibitor’s inhibition effectiveness and corrosion behavior. The results presented in Table 3 indicate a clear trend towards increased inhibition efficiency with higher concentrations of the inhibitor (Al-Amiery et al., 2023; Yadav et al., 2023b; Narang et al., 2023). For instance, at 50 ppm concentration, the, I.E., % is recorded at 61.37%, demonstrating a substantial lessen in the C_R_ compared to the uninhibited condition. As the inhibitor concentration rose to 500 ppm, the, I.E., % value significantly rose to 97.35%, indicating a remarkable improvement in corrosion protection. Furthermore, the values of R_ct_ offer valuable info about the potency of the inhibitor in impeding the corrosion process. A higher R_ct_ value of 395.55 Ωcm^2^ corresponds to lower corrosion activity, indicating that the inhibitor successfully impedes the corrosion-causing charge transfer processes. The observed increase in R_ct_ with rising inhibitor concentration corroborates the enhanced inhibition efficiency observed in the, I.E., % values. The values of R_s_ across different inhibitor concentrations reveal notable variations. For instance, at 50 ppm of Prinivil, R_s_ is recorded at 1.673 Ω, indicating relatively low resistance to ionic flow. As the concentration of Prinivil increases to 500 ppm, R_s_ increases to 4.879 Ω(Alamry et al., 2023; Fawzy et al., 2023b). This elevation in Rs suggests reduced ionic conductivity of the solution, which could facilitate reduction in the electrochemical processes at the metal-electrolyte interface, which results in enhanced corrosion inhibition.

**TABLE 3:** IE% values for the mild steel’s corrosion inhibition in corrosive media exposed to 50–500 ppm of inhibitor at 298 K.

Conc. (ppm)	CPE.Yo (µF)	*N*	R_s_ (Ω)	R_p_(Ω)	Rct _in_ (Ωcm^2^)	Rct _bl_ (Ωcm^2^)	IE%	ɵ	χ^2^
Blank	422.78	0.965	0.984	3.814	-	10.47	-	-	0.579
50	248.12	0.819	1.67	23.946	27.11	10.47	61.37	0.114	0.114
100	247.58	0.798	1.379	30.566	33.52	10.47	68.76	0.136	0.136
200	232.25	0.808	1.399	38.283	43.61	10.47	75.99	0.166	0.166
300	163.94	0.709	1.935	153.42	166.80	10.47	93.72	0.192	0.192
500	235.54	0.58	4.879	398.28	395.55	10.47	97.35	0.564	0.564

In contrast, R_p_ values demonstrate the corrosion resistance of the metallic surface in the existence of Prinivil. At 50 ppm, R_p_ is measured at 23.946 Ω, indicating a moderate level of polarization resistance. As the concentration of Prinivil increases, R_p_ also rises steadily, reaching 398.28 Ω at 500 ppm. These increasing R_p_ values suggest improved corrosion resistance conferred by the inhibitive layer formed by Prinivil upon the metallic surface. Additionally, the degree of surface coverage (ɵ) values reflect the extent to which the inhibitor molecules adhere to the surface of the mild steel, developing a defensive coating against corrosion. The data demonstrates a proportional increase in ɵ with rising inhibitor concentration, indicating greater surface coverage and adsorption of inhibitor molecules on the metallic surface (Merimi et al., 2023b; Shetty et al., 2023). Moreover, the consistency of the goodness of fit parameter (χ^2^) across different inhibitor concentrations indicates the robustness and reliability of the electrochemical measurements and data analysis techniques employed in the study. This reinforces the validity of the results and underscores the accuracy of the reported inhibition efficiencies and surface coverage values.

### 3.3 Activation parameters

Activation parameters in corrosion inhibition encompass the thermodynamic and kinetic factors pivotal for understanding the efficiency of corrosion inhibitors in mitigating the corrosion phenomenon. These parameters exhibit a crucial aspect in elucidating the inhibition mechanism and predicting inhibitor performance under varying conditions (Mandal et al., 2023; Mohapatra et al., 2023). Key activation variables include activation energy (E_a_), entropy of activation (ΔS) and enthalpy of activation (ΔH). E_a_ of the corrosion mechanism under various temperature conditions was determined using Equation 15, both with and without the inhibitor.

$$\ln C_{R}=lnA-\frac{E_{a}}{RT}$$ (15)

**FIGURE 6:**
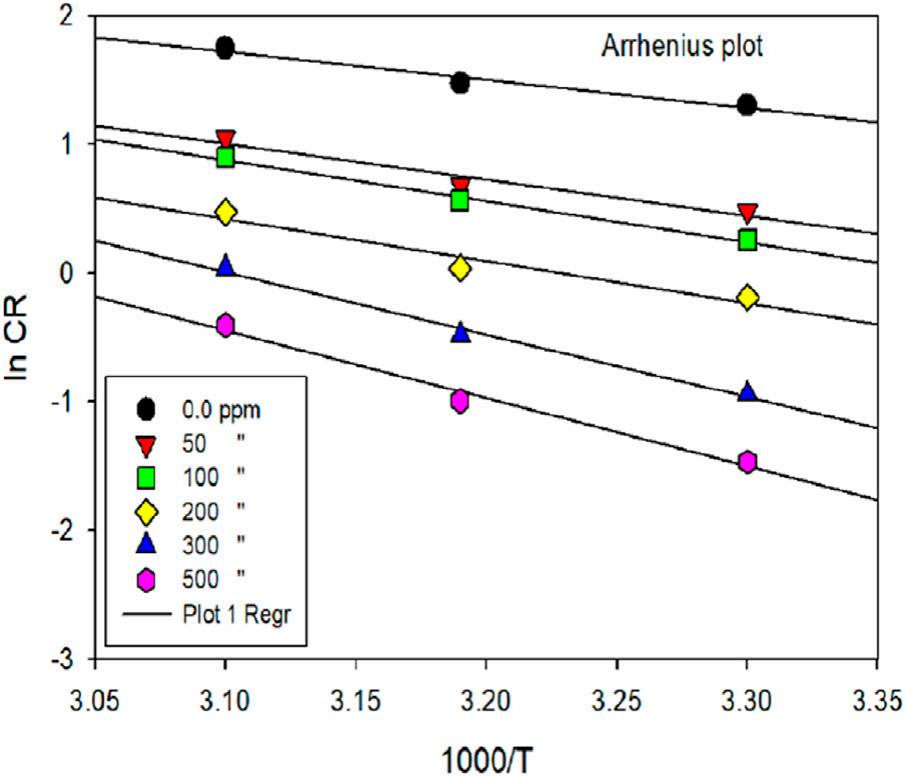
ln [C_R_] vs. 1,000/T for mild steel corrosion in 1 M HCl media in the exclusion and inclusion of various dosages of inhibitor.

The thermodynamic variables for the corrosion phenomenon, ΔH and ΔS, were computed using the transition state concept. The slope of the eqn. (-E_a_/R) was utilized to calculate the E_a_ of the metal-dissolving mechanism, both with and without the investigated inhibitor, as depicted in Figure 6.

$$\left.\ln\left(\frac{C_{R}}{T}\right)=\left[\left(\ln\;\left(\frac{R}{Nh}\right)\right)+\left(\frac{\Delta S}{R}\right)\right]-\frac{\Delta H}{RT}\right)$$ (16)

In this context, R, h, T, N, ΔS and ΔH represent the system’s gas constant, Planck’s constant, temperature, Avogadro’s number, entropy and enthalpy, correspondingly. Table 4 displays various activation parameters in the exclusion and inclusion of the prinivil.

**TABLE 4:** The activation variables of mild steel (E_a_, ΔH, ΔS) in the presence or absence of inhibitor.

Conc. (ppm)	Ea (kJ/mol)	ΔH (kJ/mol)	ΔS (J/mol K)	Ea- ΔH (kJ/mol)
0	18.42	15.77	−182.33	2.65
50	23.18	20.53	−173.55	2.65
100	26.55	23.90	−164.16	2.65
200	27.16	24.51	−166.05	2.65
300	40.56	37.91	−127.94	2.65
500	44.14	41.49	−120.63	2.65

**FIGURE 7:**
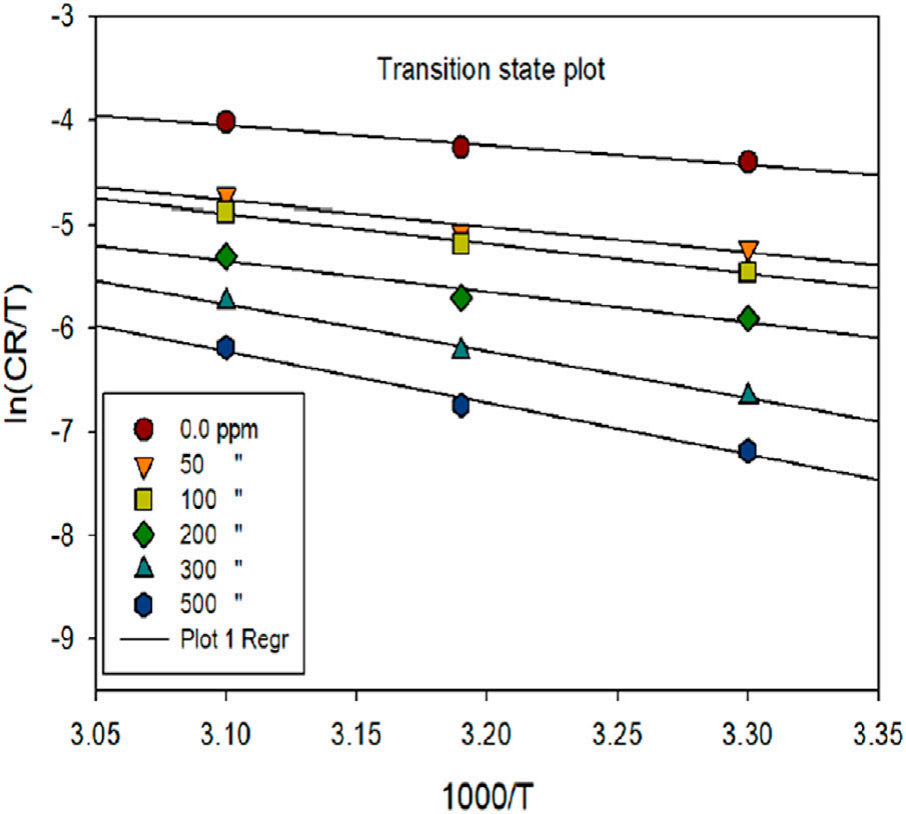
ln [C_R_/T] vs 1,000/T for mild steel corrosion in 1 M HCl media in the exclusion and inclusion of various dosages of inhibitor.

Table 4 provides activation parameters (E_a_, ΔH, ΔS) for the corrosion of mild steel in the presence and absence of prinivil, at various concentrations as shown in Figure 7. For E_a_, the values increased from 18.42 kJ/mol (0 ppm) to 44.14 kJ/mol (500 ppm) (Chowdhury et al., 2023; Katagiri et al., 2023; Zhang et al., 2023). In this context, an elevation in E_a_ values with rising inhibitor dosage suggests that the inhibitor impedes the corrosion process by enhancing the energy barrier for corrosion initiation. This implies that a higher concentration of inhibitor leads to a more significant hindrance to the corrosion reaction, thereby enhancing corrosion inhibition efficiency. Similarly, ΔH shows an increasing trend with inhibitor concentration. The values rise from 15.77 kJ/mol (0 ppm) to 41.49 kJ/mol (500 ppm). The ascending pattern of ΔH values in relation to inhibitor dosage suggests that the inhibitor alters heat exchange during the corrosion procedure. This modification in enthalpy suggests that the inhibitor molecules either absorb or release more heat compared to the uninhibited corrosion process, which could signify a modification in the corrosion phenomenon or the formation of inhibitor-metal complexes.

In contrast, ΔS exhibits a decreasing trend with increasing inhibitor concentration. The values decrease from −182.33 J/mol K (0 ppm) to −120.63 J/mol K (500 ppm) (Martinović et al., 2023). The decreasing trend of ΔS values with increasing inhibitor concentration implies that the existence of the inhibitor reduces the disorderliness associated with the corrosion phenomenon. This decrease in entropy indicates that the inhibitor molecules most likely attach to the metal surface in an organized fashion, creating a barrier that prevents corrosive species from moving freely and lessens the corrosion process’ overall unpredictability. The attained outcomes in activation parameters suggest that the inhibitor molecules modify the energy barrier and thermodynamic factors of the corrosion process, leading to increased resistance to corrosion with higher inhibitor concentrations.

### 3.4 Adsorption studies

**FIGURE 8:**
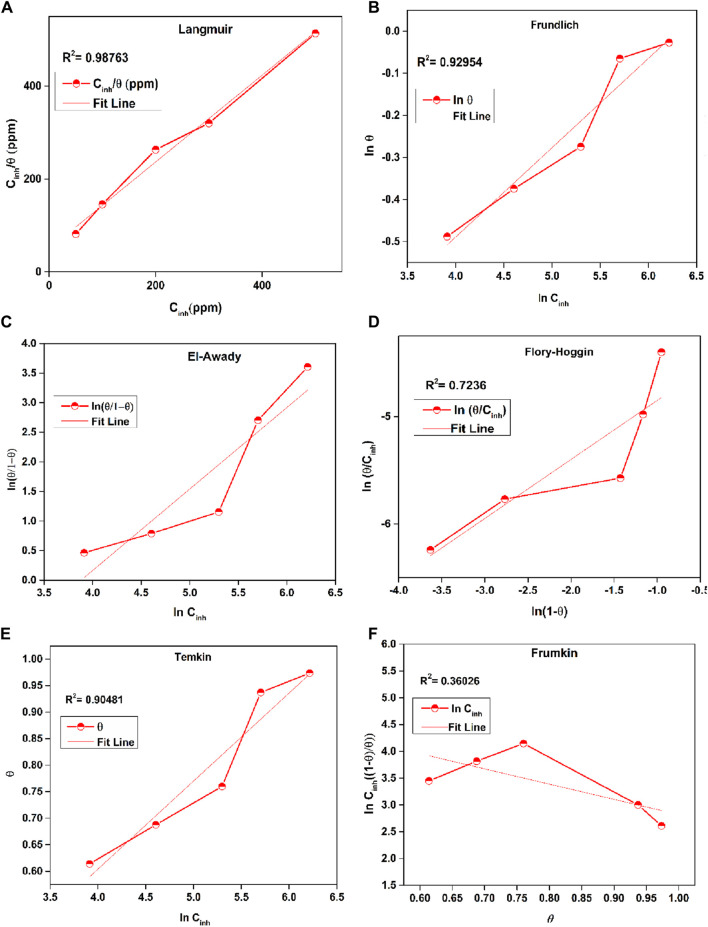
Plots of **(A)** Langmuir, **(B)** Frundlich, **(C)** El-Awady Freundlich, **(D)** Flory-Huggins, **(E)** Temkin and **(F)** Frumkin for the adsorption of inhibitor upon mild steel in 1 M HCl.

The examination of corrosion-inhibiting properties foremost depends on the examination of adsorption kinetics and isotherms, offering vital information into the adsorption properties and adherence of the inhibitor molecules upon the surfaces of deteriorated metals. Adsorption isotherms depict the association among the integrity of a corrosion inhibitor absorbed onto the deteriorated material’s substrate and its content in the media (Barrodi et al., 2023; Solmaz et al., 2023). The form of an adsorption isotherm may expose insights about the adsorption mechanism and the dynamics of the inhibitor-corroded material relationship. However, adsorption kinetics parameters define the degree to which corrosion inhibitors are assimilated onto the surface of the corroded metal. In this examination, a range of adsorption isotherms, including Langmuir, Temkin, Frumkin, Freundlich, Flory-Huggins and El-Awady Eqs 17–(22 (indicated in Figure 8), were utilized to evaluate the sorption behavior and determine the association between ϴ and inhibitor concentration (C_inh_). Examining the σ values from Table 5 helped to more effectively understand the behavior of inhibitor adsorption.

$$\frac{C_{inh}}{\theta }=\frac{1}{K_{ads}}+C_{inh}\text{ Langmuir}$$ (17)

$$\ln\left(\frac{\theta }{1-\theta }\right)=\text{lnK}+{\text{ylnC}}_{inh}\text{ El}-\text{Awady}$$ (18)

**TABLE 5:** Several adsorption parameters for Prinivil in 1 M HCl media.

Isotherm	Inhibitor	*R* ^2^	Variable		K_ads_	ΔG^o^_ads_ (J/mol)
Langmuir	Prinivil	0.987	Slope	0.934	5.023	−38.869

with Kads=K1y

$$\ln\left(\theta \right)={\text{lnK}}_{ads}+z\ln C_{inh}\text{ Freundlich}$$ (19)

$$\ln\left(\frac{\theta }{C_{inh}}\right)={\text{lnK}}_{ads}+x\ln\left(1-\theta \right)\text{ Flory}-\text{Huggin}$$ (20)

$$\ln\left[\left(\frac{\theta }{1-\theta }\right)\times \left(\frac{1}{C_{inh}}\right)\right]=-\ln K_{ads}+2d\theta \text{ Frumkin}$$ (21)

$$\theta =-\frac{1}{2a}\ln K_{ads}-\frac{1}{2a}\ln C_{inh}\text{ Temkin}$$ (22)

In this context, C_inh_ represents the concentrations of prinivil, K_ads_ signifies the adsorption equilibrium constant, and ϴ indicates the extent of the surface covered by the inhibitor. The different isotherm plots concerning the examined inhibitor were evaluated utilizing data obtained from EIS measurements.

Eq. 17, corresponding to the inverse of the intercept of a linear slope has been utilized to determine K_ads_, while Eq. 23, linking K_ads_ to ΔG^o^_ads_ (standard free energy of the adsorption isotherm) employed (Lgaz et al., 2019).

$$\Delta G_{ads}^{o}=-RT\ln\left(K_{ads}\times 999\right)$$ (23)

Herein, 999 is the concentration of H_2_O in solution in g L^-1^, T is the absolute temperature and R denotes the universal gas constant.

Table 5 provides a detailed analysis of several adsorption parameters for Prinivil in a 1M HCl solution. The Langmuir isotherm, a widely used model for describing adsorption phenomena, was employed to investigate the adsorption behavior of Prinivil. The high coefficient of determination (*R* ^2^) value of 0.987 indicates a strong linkage between the empirical data and the theoretical predictions relying on the Langmuir model (Pour-Ali et al., 2023b; Ebunta Abeng and Chikaodili Anadebe, 2023; Kesari et al., 2023). This suggests that the Langmuir isotherm effectively defines the adsorption behavior of Prinivil in the given solution. The slope variable of the Langmuir isotherm, which represents the adsorption equilibrium constant (K_ads_), was determined to be 0.934. This value indicates the extent to which Prinivil molecules were effectively adsorbed onto the surface under investigation. A high value of K_ads_ suggests a strong affinity of Prinivil for adsorption onto the surface. Furthermore, ΔG^o^_ads_ was computed to be −38.869 J/mol. This low value indicates that the adsorption phenomenon is energetically valuable, meaning that Prinivil molecules spontaneously adsorb onto the surface in the 1 M HCl media without the need for external energy input.

### 3.5 Surface morphology assessment

#### 3.5.1 SEM

**FIGURE 9:**
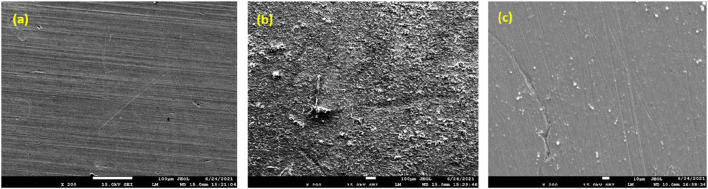
SEM images of **(A)** pristine metal, **(B)** metal dipped in 1M HCl **(C)** metal dipped in 1 M HCl + prinivil (500 ppm) at 298K for 6 hrs.

In this study, SEM analysis was conducted to examine the surface morphology and corrosion behavior of mild steel samples subjected to different conditions. After a 6 h immersion in a 1 M HCl solution at 298 K, SEM analysis was performed on three distinct samples to evaluate their corrosion characteristics. The SEM micrograph of the plain metallic sample (Figure 9A) displayed a relatively smooth surface, indicating minimal corrosion in the absence of any inhibiting agent (Nesane et al., 2023). In contrast, the SEM analysis of the metallic sample (Figure 9B) immersed solely in the 1 M HCl solution showed significant corrosion damage, characterized by the existence of numerous corrosive pits distributed across the surface. Remarkably, the SEM micrograph of the metallic sample (Figure 9B) immersed in the 1 M HCl solution with the introduction of a 500 ppm inhibitor exhibited significantly fewer corrosive pits compared to the sample immersed in the acidic solution alone. The enhanced presence of corrosion products in Figure 9C compared to Figure 9B can be attributed to the protective action of the inhibitor. When the mild steel sample was immersed in the acidic solution alone, without the inhibitor, it was more susceptible to corrosion attack, leading to the formation of corrosion products. However, when a 500 ppm inhibitor concentration was introduced, the inhibitor formed a protective layer on the metal surface, inhibiting the corrosion process. This protective layer prevented the formation of extensive corrosion products, resulting in fewer corrosive pits and a smoother surface appearance. Therefore, the more pronounced corrosion products in Figure 9C indicate the effectiveness of the inhibitor in mitigating corrosion and preserving the integrity of the metal surface (Bedair et al., 2022; Sanni et al., 2022). The inclusion of the prinivil led to a notable reduction in the formation of corrosive pits, underscoring its potential for corrosion protection in acidic environments.

#### 3.5.2 EDX analysis

The EDX analysis revealed differences in the elemental composition of the corrosion products between the blank sample and the sample treated with Prinivil (Kaya et al., 2023a; 2023b; Arifa Farzana et al., 2023; Sharma et al., 2023a; Dhonchak and Agnihotri, 2023). The EDX analysis depicted in Supplementary Figure S3 illustrates the elemental composition of mild steel samples subjected to different environments. In the sample immersed **(a)** in 1 M HCl solution, Fe dominates at 87.31 wt%, consistent with mild steel composition, accompanied by C at 6.19 wt% and O at 6.84 wt%, indicating surface oxidation typical of acidic environments. Conversely, in the sample treated **(b)** with 500 ppm Prinivil in 1 M HCl solution, iron remains dominant at 87.80 wt%, with increased carbon (8.01 wt%) and slightly reduced oxygen (5.44 wt%). Notably, nitrogen (4.04 wt%) is detected in the Prinivil-treated sample, indicating the presence of the inhibitor on the metal surface (same as mentioned in Table 6) (Thakur et al., 2022; 2023a; 2023b; Sharma et al., 2023b). This suggests adsorption or chemical bonding of Prinivil molecules, contributing to a protective layer that inhibits corrosion. The higher carbon and oxygen content in the treated sample may imply the formation of a more stable oxide layer, enhancing corrosion resistance.

**TABLE 6:** Elemental composition analysis of mild steel samples immersed in 1 M HCl solution, with and without Prinivil inhibitor (500 ppm), as determined by EDX analysis.

Element	Sample (a) (1 M HCl)	Sample (b) (1 M HCl + prinivil 500 ppm)
Fe	87.31 wt%	87.80 wt%
C	6.19 wt%	8.01 wt%
O	6.84 wt%	5.44 wt%
N	-	4.04 wt%

#### 3.5.3 Contact angle (CA) analysis

The contact angle analysis is a crucial procedure for observing the surface attributes of materials and understanding their interactions with liquids. In this study, contact angle measurements were conducted following 6 h of dipping in 1M HCl media, both with and without the existence of a corrosion inhibitor. The sample (Supplementary Figure S4A) illustrates the contact angle measurement of the pristine polished metal before dipping into the blank solution with an angle of 98.24°. The sample dipped in 1M HCl media (Supplementary Figure S4B) exhibited a contact angle of 49.64°. This relatively low contact angle indicates a moderate wetting behavior, suggesting that the acid solution interacts relatively strongly with the surface of the material (Alotaibi et al., 2022; Naggar et al., 2022; Quadri et al., 2022). On the other hand, the sample immersed in 1M HCl media containing 500 ppm of the inhibitor (Supplementary Figure S5C) revealed a significantly higher contact angle of 84.27°. This substantial increase in contact angle indicates a pronounced change in surface properties, likely attributed to the existence of the corrosion inhibitor. The higher contact angle suggests improved hydrophobicity or reduced surface wettability in the inclusion of the inhibitor, indicating the emergence of a defensive coating upon the material’s surface. This observation aligns with the corrosion inhibition efficacy results attained from other analytical techniques in the study.

### 3.6 Theoretical analysis

#### 3.6.1 DFT results

**FIGURE 10:**
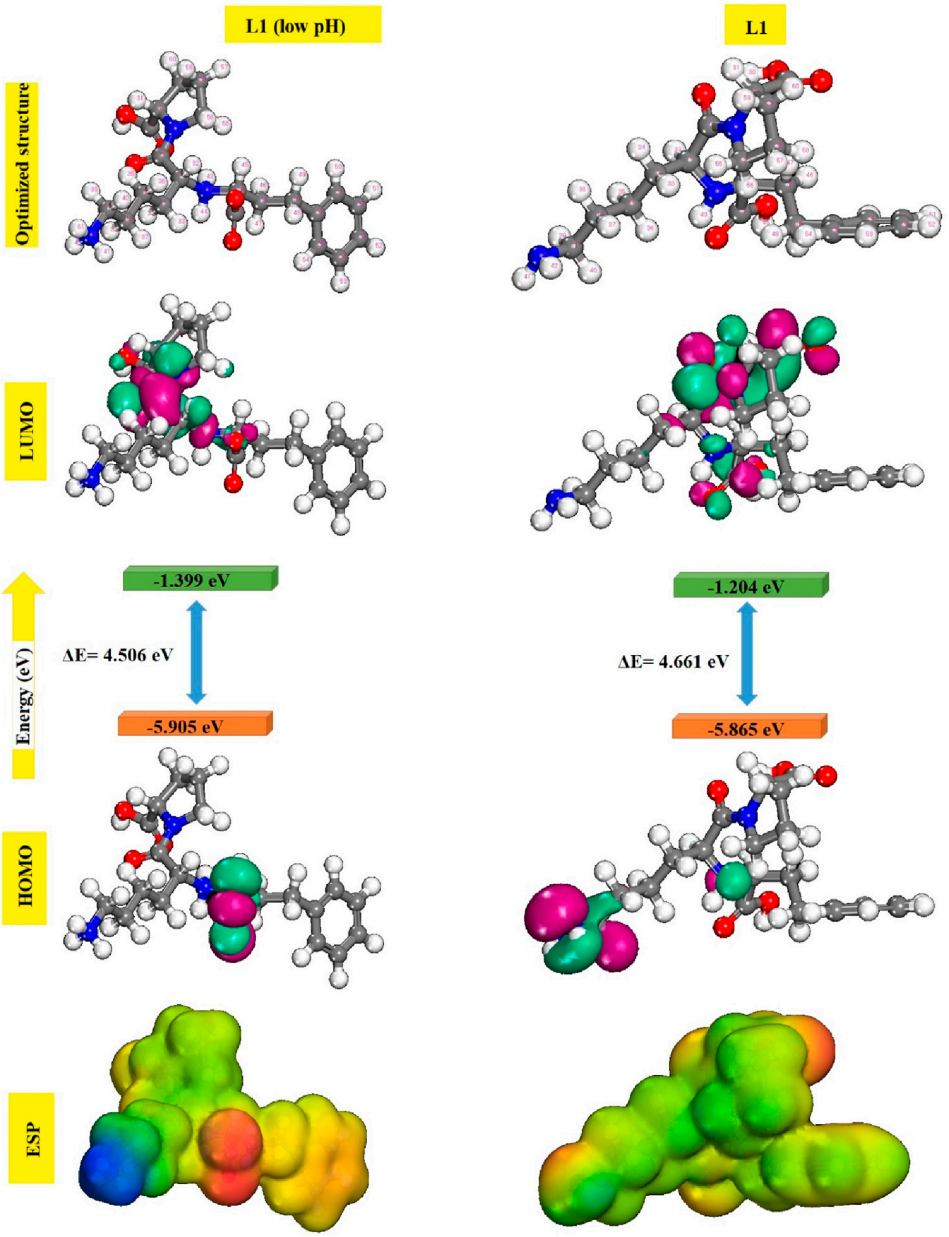
Optimized geometries, HOMO, LUMO and MEP images for prinivil (low pH) and prinivil molecules.

It is well documented in the literature that heteroatom-containing compounds such as nitrogen and oxygen in their molecular structures are often excellent corrosion inhibitors. Indeed, these compounds can be adsorbed by their free doublets onto the metallic surface, blockage of active sites and therefore reduction of corrosion rate. The efficiency of molecules is linked to their electronic properties: electron density on atoms and the character of HOMO and LUMO orbitals. According to Fukui’s theory, the HOMO and LUMO boundary molecular orbitals could be utilized to predict the reactivity of the inhibitor toward the metallic surface. This theory is based on the principle that only Frontier molecular orbitals are of real interest when studying a chemical reaction with Frontier control (Kalkhambkar and Rajappa, 2022; Swathi et al., 2022). HOMO energy is often linked to the molecule’s capacity to release electrons into available orbits. Conversely, LUMO offers details on the molecule’s ability to receive electrons. The gap between the inhibiting molecule’s HOMO and LUMO energy levels is another crucial metric since low energy gap values suggest effective inhibition. Figure 10 displays the electron density distributions of the HOMO and LUMO molecular orbitals prinivil (low pH) and prinivil inhibitors, as well as the molecular Frontier orbitals.

It could be observed that the electron density of the LUMO location was distributed almost throughout the molecule, because of the existence of oxygen, nitrogen, and carbon atoms, including multiple electrons of chemical configurations of inhibitors prinivil (low pH) and prinivil studied. Thus, the iron atom’s unoccupied orbit may receive electrons from inhibitory molecules to form coordination bonds. Inhibitory molecules may also accept electrons of the iron atom with its anti-binding orbitals to form bonding bounds in return (Burhagohain et al., 2022; Shao et al., 2022). ESP analysis is used as a fundamental tool to study the behavior of atoms and molecules; various non-covalent molecular interactions and properties have been well interpreted by ESP analysis. ESP mapping makes it easier to see where a molecule’s electron density is especially high or low, emphasizing the molecule’s reactive regions. Red represents the locations with the highest negative electrostatic potential, while blue indicates the regions with the highest positive electrostatic potential. As demonstrated by the ESP mappings of the chemical species under investigation in Figure 10. From the figure above, the most negative electrostatic potential is distinctly localized to atoms O and N, indicating that these atoms may be the main contributors to the coordination process with ions. The theoretical study of the prinivil (low pH) and prinivil inhibitors, allowed us to identify a number of structural and electronic parameters of the systems studied. The parameters are gathered in Table 7.

**TABLE 7:** Theoretical variables calculations for prinivil (low pH) and prinivil inhibitors.

Theoretical variables	Prinivil (low pH)	Prinivil
I (eV)	5.905	5.865
A (eV)	1.399	1.204
χ (eV)	3.652	3.534
η (eV)	2.253	2.330
σ (eV^-1^)	0.444	0.429
∆N	0.560	0.276
∆E_back-donation_	−0.563	−0.582

*E*_HOMO_ values evolve in the direction: prinivil > prinivil (low pH), which explains why the prinivil molecule has a higher electron-donating capacity than prinivil (low pH). While *E*_LUMO_ follows the order prinivil (low pH) <prinivil, this means that prinivil (low pH) has greater electron acceptor capacity than prinivil. Additionally, the prinivil (low pH) molecule is softer (σ = 0.444) and possesses the minimum ΔE value (4.506 eV), that facilitates its adsorption on the substrate and thus increases its, I.E.,%. Moreover, the theoretical value of iron (χ_Fe_ = 4.82 eV and η_Fe_ = 0) is utilized for calculating the proportion of inhibitor ΔN on the surface of MS(Kucharski et al., 2022; Said et al., 2022). The, I.E., % that results from electron donation is related to the ΔN values. Lukovit’s research indicates that when ΔN is less than 3.6, the, I.E., % rises along with the capacity to contribute electrons to the metallic surface. In our research, the prinivil (low pH) and prinivil presented values below 3.6 following the order prinivil (low pH) > prinivil and therefore, the latter are electron donating inhibitors and the metallic substrate is an acceptor.

The computed Mulliken charges of the atoms are displayed in Supplementary Figure S5. Examination of these results shows that negatively charged atoms with high electron density behave as nucleophilic sites when engaging with the substrate, while positively charged atoms can accept electrons from the metal (Mahadevaswamy et al., 2022; Priyanka and Nalini, 2022). The values in Supplementary Figure S5 show that all nitrogen and oxygen atoms exhibit a significant excess of negative charge. Some carbon atoms are also likely to be active sites for the adsorption of the inhibitors tested to the metallic surface. As a consequence, the corrosion rate is reduced.

Fukui model indices are employed to clarify the localized reactivity of several chemical inhibitors. It follows that the atoms whereby the metal’s surface adsorption process takes place could be assumed. Accordingly, the locations with an elevated *f*_k_^+^ value are those that the *f*_k_^+^ prefers, and the places with the lowest *f*_k_^–^values are those that the *f*_k_^–^finds attractive. Table 8 comprises the Fukui indices derived from the Mulliken charges technique for prinivil (low pH) and prinivil inhibitors forms (Hameed et al., 2021; Khiev et al., 2021; Lu et al., 2021). Table 8 could be used to identify appropriate sites for the *f*_k_^+^ and *f*_k_^–^assault for the prinivil under investigation. This inhibitor is more effective in the instance of compound prinivil (low pH) than it is in the case of the prinivil inhibitor. The most effective sites are C, O and N atoms with an improved attack attribute, which are O (1), C (6), O (16), O (17) and O (26) for prinivil (low pH) and O (1), C (6), N (12) and O (26) for prinivil as listed in Table 8.

**TABLE 8:** *f*_k_^+^ and *f*_k_^–^matrics relied on the Mulliken charges procedure of prinivil (low pH) and prinivil inhibitors forms.

Atom	Prinivil (low pH)		Atom	Prinivil	
	*f*_k_^+^	*f*_k_^–^		*f*_k_^+^	*f*_k_^–^
O (1)	**0.045**	**0.037**	O (1)	**0.051**	**0.045**
C (2)	0.041	0.027	C (2)	0.040	0.027
O (3)	0.024	0.018	O (3)	0.026	0.022
C (4)	0.063	−0.010	C (4)	−0.006	−0.006
N (5)	0.013	0.015	N (5)	0.006	0.009
C (6)	**0.063**	**0.044**	C (6)	**0.040**	0.027
C (7)	−0.002	−0.005	C (7)	−0.017	−0.018
C (8)	−0.012	−0.011	C (8)	−0.006	−0.006
C (9)	−0.005	−0.004	C (9)	−0.006	−0.007
C (10)	−0.001	−0.001	C (10)	−0.005	−0.006
C (11)	−0.001	−0.000	C (11)	−0.007	−0.011
N (12)	−0.001	−0.000	N (12)	**0.051**	**0.080**
N (13)	−0.011	−0.008	N (13)	0.026	**0.042**
C (14)	−0.006	−0.008	C (14)	−0.017	−0.015
C (15)	0.019	0.025	C (15)	0.047	0.033
O (16)	**0.084**	0.019	O (16)	0.021	0.017
O (17)	**0.077**	**0.109**	O (17)	0.044	0.036
C (18)	−0.013	−0.014	C (18)	−0.009	−0.009
C (19)	−0.011	−0.011	C (19)	−0.015	−0.014
C (20)	0.018	0.019	C (20)	0.016	0.016
C (21)	0.033	0.033	C (21)	0.034	0.031
C (22)	0.031	0.029	C (22)	0.029	0.026
C (23)	0.032	0.032	C (23)	0.031	0.031
C (24)	0.029	0.028	C (24)	0.032	0.028
C (25)	0.034	0.033	C (25)	0.021	0.020
O (26)	**0.064**	**0.059**	O (26)	**0.051**	**0.051**
C (27)	−0.013	−0.011	C (27)	−0.009	−0.009
C (28)	−0.002	−0.001	C (28)	−0.006	−0.005
C (29)	−0.003	−0.002	C (29)	−0.005	−0.004
H (30)	0.012	0.009	H (30)	0.011	0.009
H (31)	0.032	0.026	H (31)	0.033	0.028
H (32)	0.026	0.021	H (32)	0.036	0.040
H (33)	0.012	0.010	H (33)	0.013	0.014
H (34)	0.017	0.016	H (34)	0.015	0.016
H (35)	0.007	0.007	H (35)	0.009	0.011
H (36)	0.008	0.007	H (36)	0.007	0.008
H (37)	0.004	0.004	H (37)	0.007	0.010
H (38)	0.004	0.004	H (38)	0.010	0.013
H (39)	0.002	0.002	H (39)	0.012	0.018
H (40)	0.002	0.002	H (40)	0.019	0.030
H (41)	0.002	0.001	H (41)	0.013	0.020
H (42)	0.002	0.001	H (42)	0.013	0.020
H (43)	0.018	0.016	H (43)	0.021	0.023
H (44)	0.020	0.019	H (44)	0.033	0.031
H (45)	0.026	0.030	H (45)	0.013	0.011
H (46)	0.021	0.024	H (46)	0.023	0.024
H (47)	0.018	0.020	H (47)	0.003	0.004
H (48)	0.018	0.019	H (48)	0.019	0.018
H (49)	0.018	0.020	H (49)	0.024	0.023
H (50)	0.025	0.025	H (50)	0.025	0.024
H (51)	0.024	0.025	H (51)	0.024	0.023
H (52)	0.025	0.025	H (52)	0.025	0.024
H (53)	0.024	0.024	H (53)	0.025	0.024
H (54)	0.025	0.025	H (54)	0.025	0.024
H (55)	0.021	0.018	H (55)	0.025	0.024
H (56)	0.018	0.016	H (56)	0.008	0.006
H (57)	0.013	0.011	H (57)	0.016	0.015
H (58)	0.011	0.010	H (58)	0.005	0.005
H (59)	0.012	0.010	H (59)	0.014	0.013
H (60)	0.014	0.012	H (60)	0.014	0.013
H (61)	0.002	0.001			

#### 3.6.2 MC and MD simulations

**FIGURE 11:**
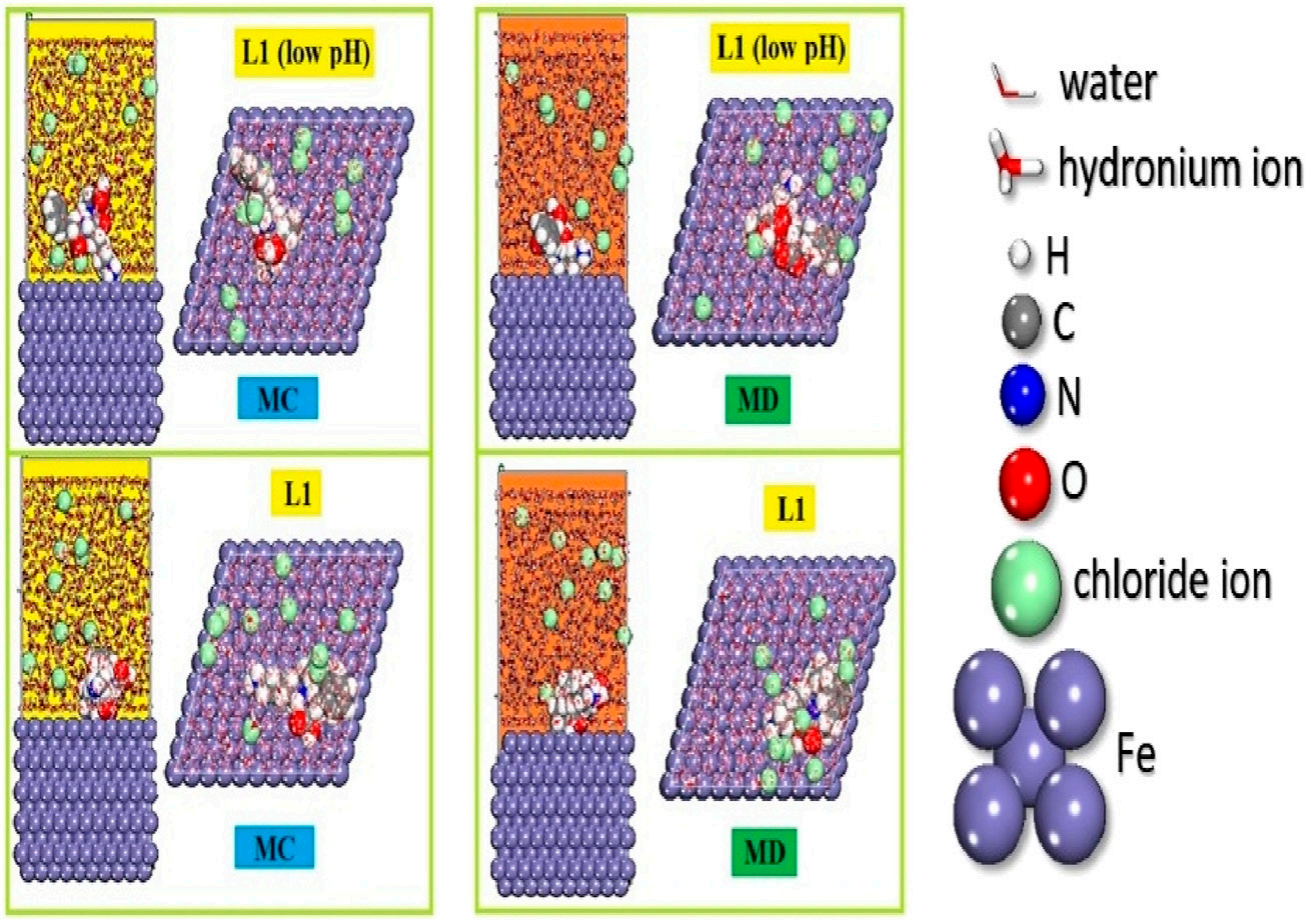
MC and MD simulations provide adsorption setups and prinivil (L1) (low pH) and prinivil inhibitor positions.

Monte Carlo (MC) and Molecular Dynamics (MD) simulations are broadly employed in scientific research to explore the interactions between inhibitory molecules and metallic surfaces. These computational techniques offer valuable insights into the equilibrium adsorption configurations of inhibitors on metal surfaces and their inhibitory effectiveness (Abdollahi et al., 2020; Ma et al., 2020; Raghavendra, 2020). In a specific case involving prinivil (low pH) and prinivil inhibitors on the Fe (1 1 0) surface, both MC and MD simulations reveal that the inhibitors adopt a nearly plane-parallel orientation upon adsorption (Figure 11). This orientation proves advantageous as it facilitates efficient adsorption onto the carbon steel surface, effectively blocking a maximum number of active sites. Consequently, this configuration enhances the inhibitory efficacy of the inhibitors by providing extensive surface coverage and minimizing the potential for corrosion or other detrimental processes. MC and MD simulations make a substantial contribution to the comprehension of corrosion inhibition processes and facilitate the development of more potent inhibitors by elucidating the behavior of inhibitory compounds on metallic surfaces. The adsorption energies for the prinivil (low pH) and prinivil compounds are displayed in Supplementary Figure S6. The energy produced during the adsorption of the relaxed components adsorbed onto the substrate is the cause of the E_ads_. The adsorbent component’s stiff strain and adsorption energies add up to the E_ads_. A higher interaction between an inhibitory molecule and a Fe (1 1 0) is indicated by high negative Eads values. These findings indicate that the inhibitory potency of the chosen chemicals is arranged as follows: prinivil (low pH) (*E*_ads_ = −287.35 kcal/mol) > prinivil (*E*_ads_ = −152.95 kcal/mol).

**FIGURE 12:**
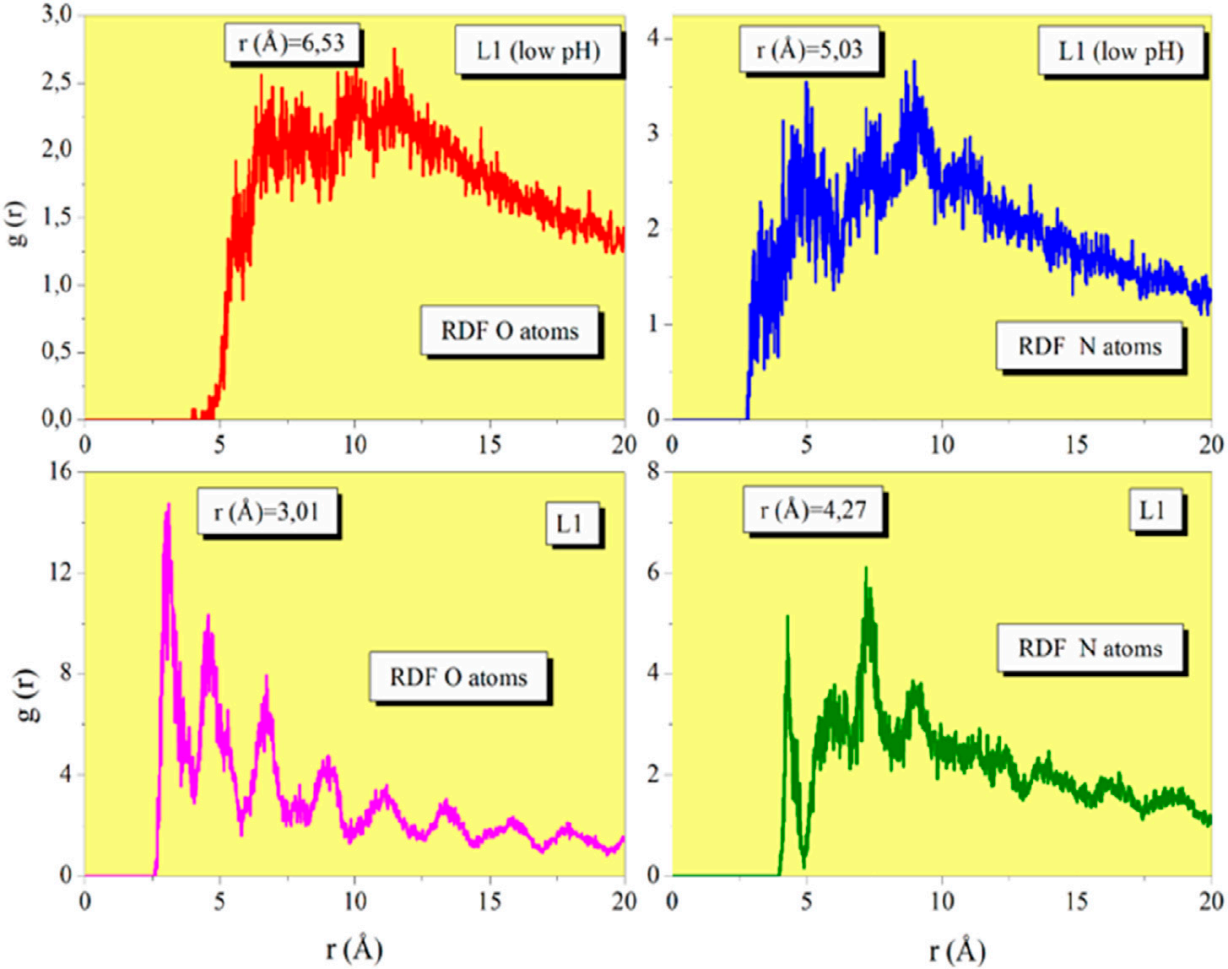
RDF of the O and N heteroatoms for prinivil (low pH) and prinivil obtained via MD simulations.

The radial distribution function (RDF) in MD simulations is a useful tool for surface-molecule interaction analysis. References demonstrate how extensively the RDF (shown in Figure 12), commonly referred to as “g,” has been studied in the literature (Chauhan et al., 2018; Soltaninejad and Shahidi, 2018). The RDF peak for physisorption is larger than 3.5 Å, while the pinnacle for chemisorption is between 1 and 3.5 Å. As demonstrated, the proximity of prinivil (low pH) and prinivil compounds to the metallic surface shows that both inhibitors and the metallic surface have a reasonably strong connection. This corroborates the inhibitors’ reflected inhibitory efficiency.

## 4 Adsorption mechanism

Prinivil, a potent corrosion inhibitor for mild steel in a corrosive environment, operates through a sophisticated adsorption mechanism comprising physisorption and chemisorption processes as depicted in Supplementary Figure S7. Initially when introduced into the acidic medium, prinivil molecules adhere to the metal surface primarily through weak intermolecular forces like dipole-dipole interactions and van der Waals, characteristic of physisorption (Deyab et al., 2022; Ikeuba et al., 2022). While this initial layer offers some degree of protection against corrosion, its stability and longevity may be limited, especially in harsh and corrosive environments. Moreover, the potency of prinivil as a corrosion inhibitor significantly amplifies through chemisorption, a more robust and stable adsorption process. During chemisorption, prinivil molecules form robust and specific chemical bonds with the metallic substrate. These bonds are characterized by their durability and resistance to displacement by aggressive corrosive agents present in the acidic solution. Prinivil’s molecular structure plays a crucial role in facilitating this chemisorption process. Its composition includes functional moieties including oxygen and nitrogen, which readily engage in chemical interactions with the metallic surface. Moreover, the presence of aromatic rings and electron-rich groups in prinivil’s molecular structure further enhances the strength and stability of chemisorption. These features enable prinivil to establish a dense and defensive layer upon the metallic surface, effectively shielding it from corrosive attack over extended periods.

Additionally, Table 9 presents a comparative evaluation of various drug compounds as corrosion inhibitors for mild steel in corrosive solutions, highlighting the novelty of our investigated inhibitor, prinivil (Arifa Farzana et al., 2023; Sharma et al., 2023b). Among the listed inhibitors, prinivil, tested in a 1M HCl media at a concentration of 500 ppm, exhibits a remarkable maximal, I.E.,% of 97.35%. This finding underscores the efficacy of prinivil as a corrosion inhibitor, surpassing the inhibitory performance of other drugs such as terazosin, mebendazole, clonazepam, and others, as reported in the references provided. The high inhibition efficiency of prinivil positions it as a viable candidate for corrosion inhibition in various industrial applications.

**TABLE 9:** An assessment of the effectiveness of various drugs in preventing mild steel corrosion in a corrosive medium.

S. No.	Drugs	Corrosive media	Dosage	IE% achieved (%)	Ref.s
1	Terazosin	1 M HCl	150 ppm	94.8	Shoair et al. (2024)
2	Mebendazole	1 M HCl	5 mM	94.4	Han et al. (2023)
3	Clonazepam	3.5 wt% NaCl	500 ppm	91.4	Abeng et al. (2023)
4	Acetaminophen	1 M HCl	5 × 10^−3^(M)	85	Merimi et al. (2023b)
5	Kanamycin	0.5 M HCl	1,000 ppm	80	Shetty et al. (2023)
6	Bifonazole	1 M HCl	375 mg L^−1^	92.08	Abdallah et al. (2023)
7	Terconazole	1 M HCl	375 mg L^−1^	94.19	Abdallah et al. (2023)
8	Tizanidine	10% HCl	7 × 10^−3^ M	97.1	Iroha et al. (2023)
9	Ampicillin	5% HCl	20 mM	96.7	Alamry et al. (2023)
10	Prinivil	1 M HCl	500 ppm	97.35	Currently investigated inhibitor

## 5 Conclusion

The combined experimental and computational approach presented in this study offers crucial information into the corrosion inhibition attributes of prinivil in acidic environments. The findings open the door for more investigation into corrosion inhibition and aid in the creation of efficient corrosion protection strategies. These findings are as follows:

1. The electrochemical investigation, including PDP and EIS, revealed a significant reduction in corrosion current densities (i_corr_) with increasing concentrations of Prinivil. For instance, at 500 ppm concentration, i_corr_ decreased to 92.77 µA, indicating a high corrosion inhibition efficiency.
2. Gravimetric measurements showed a consistent decrease in the C_R_ of mild steel with increasing concentrations of Prinivil. At 500 ppm concentration, the C_R_ dropped to 0.23 mm/y, demonstrating the effectiveness of Prinivil in mitigating corrosion.
3. The evaluation of activation variables, including enthalpy (ΔH), entropy (ΔS) and activation energy (E_a_), revealed a trend towards higher inhibition efficiency with increasing concentrations of Prinivil. For instance, at 500 ppm concentration, E_a_ increased to 43.0245 kJ/mol, indicating a stronger interaction between Prinivil and the metal surface.
4. Adsorption isotherms, particularly the Langmuir model, provided insights into the adsorption behavior of Prinivil upon the metallic substrate. The Langmuir adsorption isotherm exhibited a high regression coefficient (*R* ^2^ = 0.987) and a K_ads_ value of 0.93448 at 500 ppm concentration, indicating favorable adsorption characteristics.
5. Molecular dynamics (MD) and Monte Carlo (MC) simulations validated Prinivil’s atomic-level stability over the metallic surface. These computations validated the experimental results by offering a molecular explanation of the interactions between Prinivil molecules and the metallic surface.

## Data Availability

The original contributions presented in the study are included in the article/Supplementary Material, further inquiries can be directed to the corresponding author.
