# Supplementary material for: Unraveling the corrosion inhibition behavior of prinivil drug on mild steel in 1M HCl corrosive solution: insights from density functional theory, molecular dynamics, and experimental approaches
Source: Front Chem. 2024 Jun 13;12:1403118. doi: 10.3389/fchem.2024.1403118 (PMC11212477; doi:10.3389/fchem.2024.1403118)
Supplement: Supplementary file 1 [file DataSheet1.pdf]

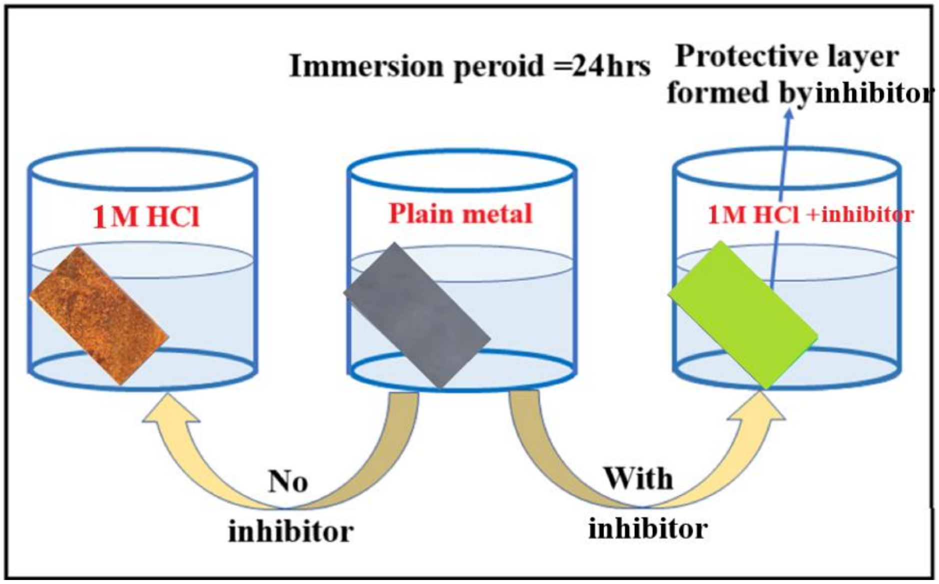

Fig. S1: Diagrammatic depiction of the investigation of weight loss.

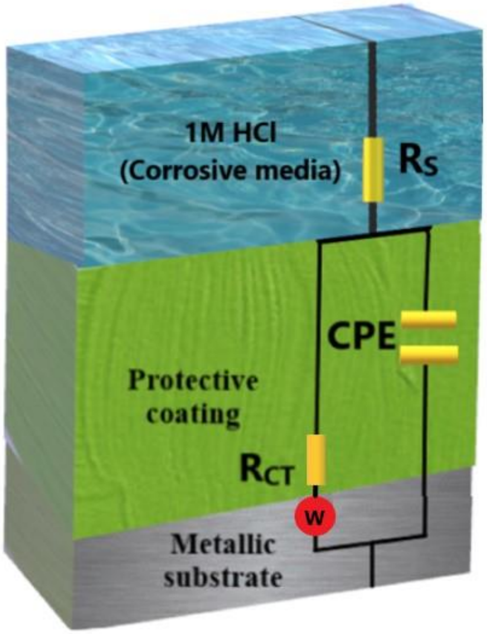

Fig. S2: An equivalent circuit employed for the fitting of the data.

(a)

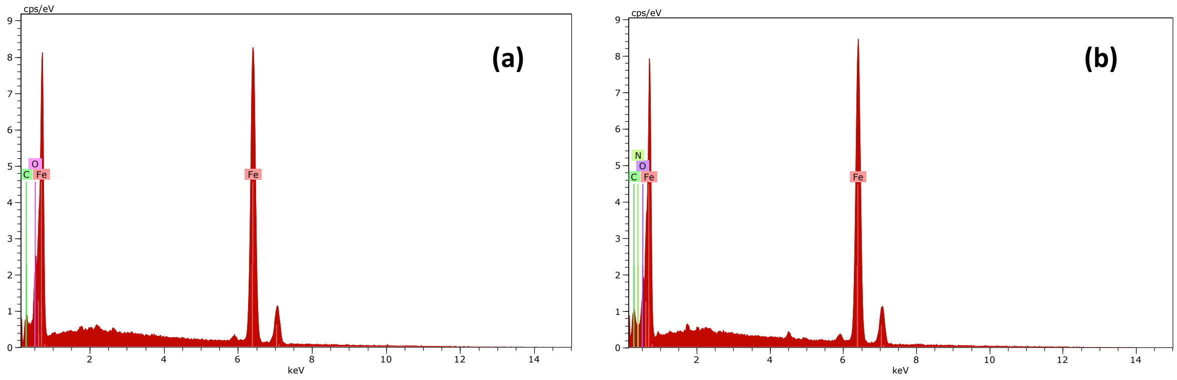

Fig. S3: EDX analysis of **(a)** metal dipped in 1M HCl **(b)** metal dipped in 1 M HCl + prinivil (500ppm) at 298K for 6hrs.

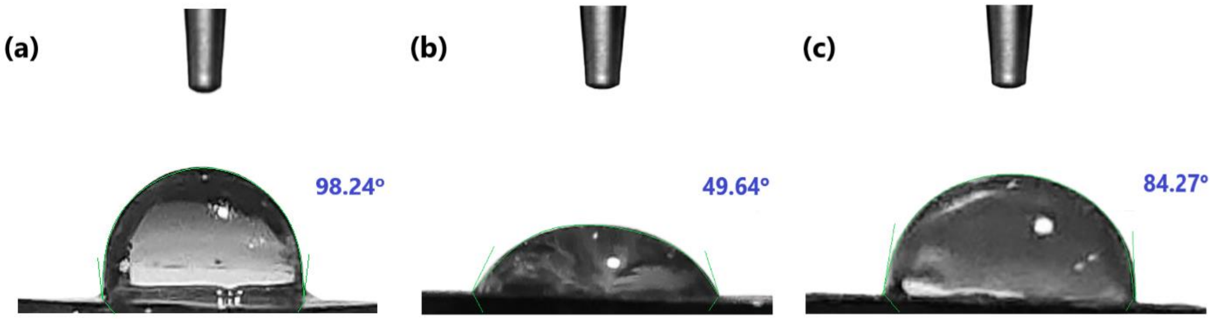

Fig. S4: Contact angle measurement of the **(a)** pristine metallic specimen **(b)** metallic specimen immersed in 1 M HCl media **(c)** metallic specimen immersed in 1 M HCl solution with 500 ppm inhibitor.

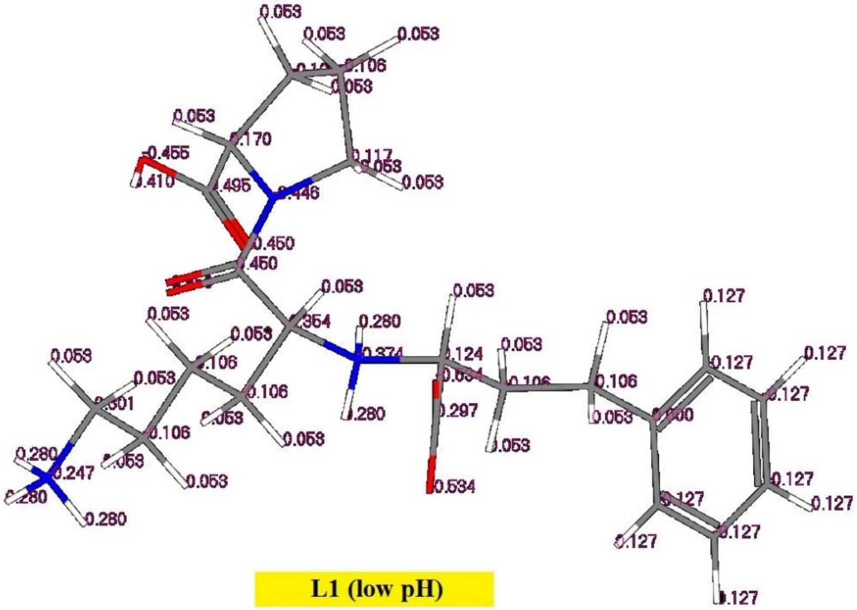

L1 (low pH)

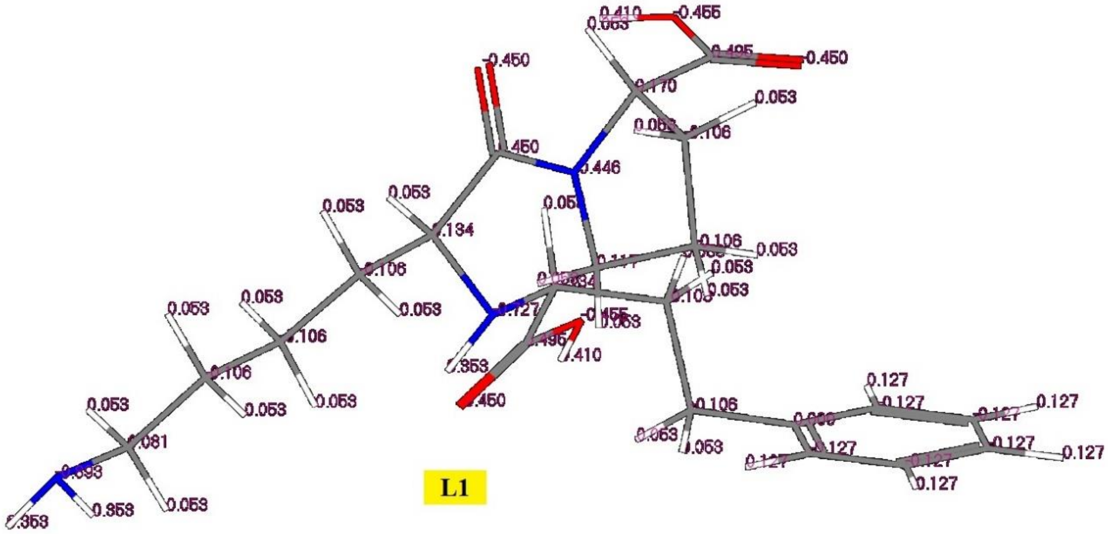

L1

Fig. S5: Distribution of MAC values for atoms C, O and N of prinivil (L1) (low pH) and prinivil inhibitors forms.

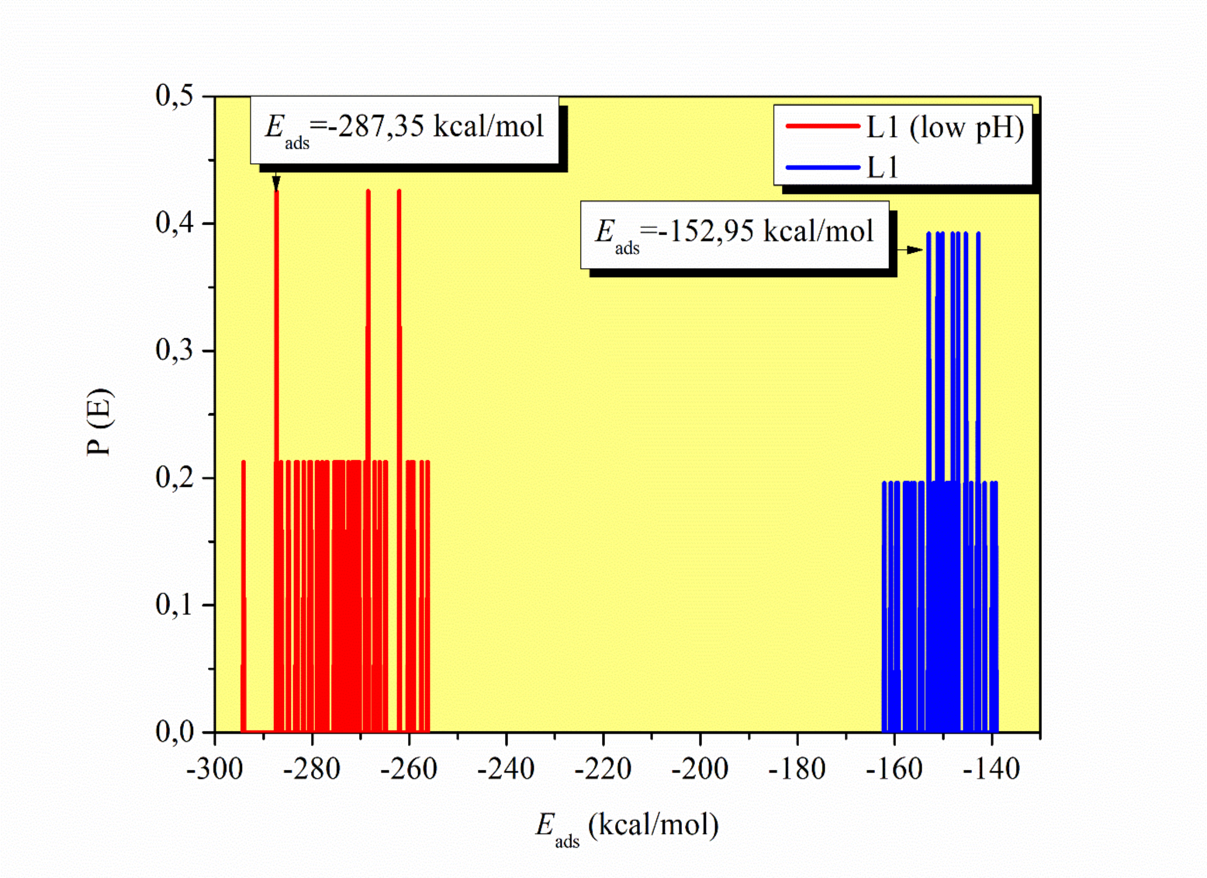

Fig. S6: E_{ads} distribution for prinivil (low pH) and prinivil inhibitors by MC simulation.

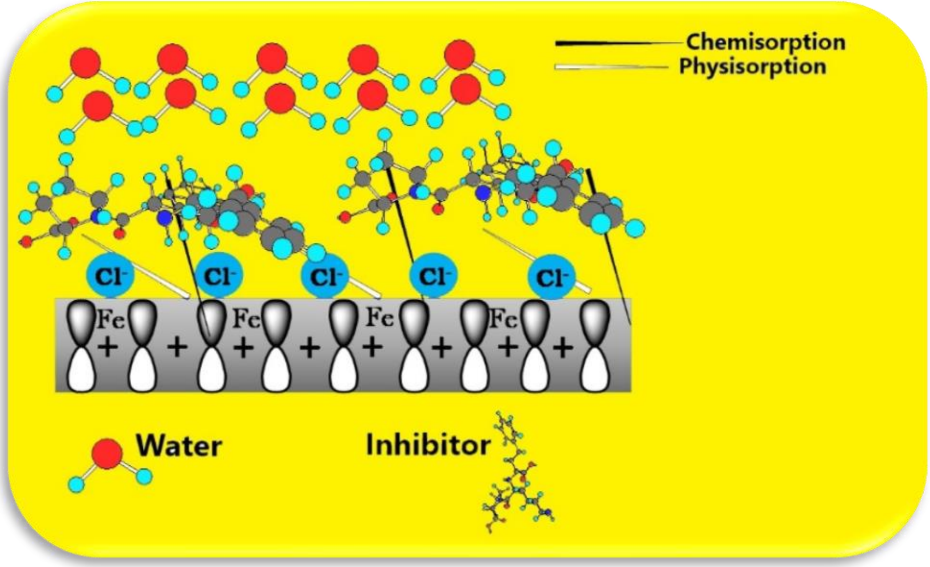

Fig. S7: Illustration of the adsorption mechanism of the inhibitor on the metallic substrate.
